# Hydrogen Bond Blueshifts in Nitrile Vibrational Spectra Are Dictated by Hydrogen Bond Geometry and Dynamics

**DOI:** 10.1021/jacsau.4c00811

**Published:** 2024-12-05

**Authors:** Jacob M. Kirsh, Jacek Kozuch

**Affiliations:** † Department of Chemistry, Stanford University, Stanford, California 94305-5012, United States; ‡ Freie Universität Berlin, Physics Department, Experimental Molecular Biophysics, Arnimallee 14, 14195 Berlin, Germany; § Freie Universität Berlin, SupraFAB Research Building, Altensteinstr. 23a, 14195 Berlin, Germany

**Keywords:** nitriles, hydrogen bonding, vibrational Stark effect, AMOEBA force field, density functional theory

## Abstract

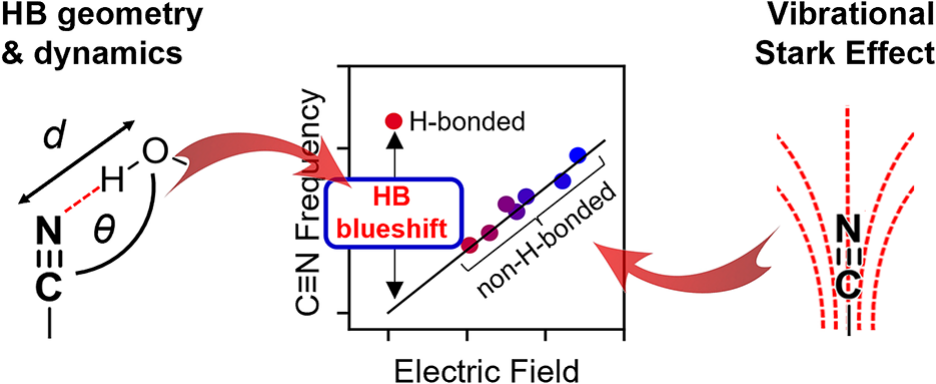

Vibrational Stark effect (VSE) spectroscopy has become one of the most important experimental approaches to determine the strength of noncovalent, electrostatic interactions in chemistry and biology and to quantify their influence on structure and reactivity. Nitriles (C≡N) have been widely used as VSE probes, but their application has been complicated by an anomalous hydrogen bond (HB) blueshift which is not encompassed within the VSE framework. We present an empirical model describing the anomalous HB blueshift in terms of H-bonding geometry, i.e., as a function of HB distance and angle with respect to the C≡N group. This model is obtained by comparing vibrational observables from density functional theory and electrostatics from the polarizable AMOEBA force field, and it provides a physical explanation for the HB blueshift in terms of underlying multipolar and Pauli repulsion contributions. Additionally, we compare predicted blueshifts with experimental results and find our model provides a useful, direct framework to analyze HB geometry for rigid HBs, such as within proteins or chemical frameworks. In contrast, nitriles in highly dynamic H-bonding environments like protic solvents are no longer a function solely of geometry; this is a consequence of motional narrowing, which we demonstrate by simulating IR spectra. Overall, when HB geometry and dynamics are accounted for, an excellent correlation is found between observed and predicted HB blueshifts. This correlation includes different types of nitriles and HB donors, suggesting that our model is general and can aid in understanding HB blueshifts wherever nitriles can be implemented.

## Introduction

Hydrogen bonds (HBs) are among the most important noncovalent interactions in chemistry and biology.^1,2^ For instance, they play a key structural role in the (self-)assembly of supramolecular complexes^3,4^ and the folding of DNA, peptides, and proteins.^5−7^ Furthermore, HBs often act as essential motifs to accelerate reactions in both organocatalytic^8−10^ and enzymatic settings.^11−13^ Despite their importance, relatively few experimental methods exist that can be used to characterize HBs within a quantitative, physical framework. One such method is vibrational Stark effect (VSE) spectroscopy, which enables the measurement of local electric field strengths of specific noncovalent interactions via changes to observables in vibrational spectra.^14^ As such, VSE spectroscopy has been used to measure electric fields in solvents,^15−17^ at electrode interfaces,^18−22^ and in membranes^23−25^ and proteins.^26−30^ The VSE describes the influence of an electric field () on a vibrational frequency (ν; in units of cm^–1^) via the dipolar VSE equation
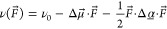
1awith the zero-field frequency ν_0_, the difference dipole , (i.e., the linear field sensitivity with its magnitude  referred to as the Stark tuning rate), and the difference polarizability Δα.^14^ Further, eq 1a is often written in linear form
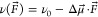
1bbecause Δα is typically experimentally negligible.^14^ Several vibrational modes, such as the carbonyl (C=O) stretch,^14,27,29,31−33^ have become very useful VSE sensors because they behave according to eq 1b.^14,34^ In this way, they have enabled the assessment of electric field strengths for HBs and other noncovalent interactions in the condensed phase.^15,16,33,35,36^

**Figure 1:**
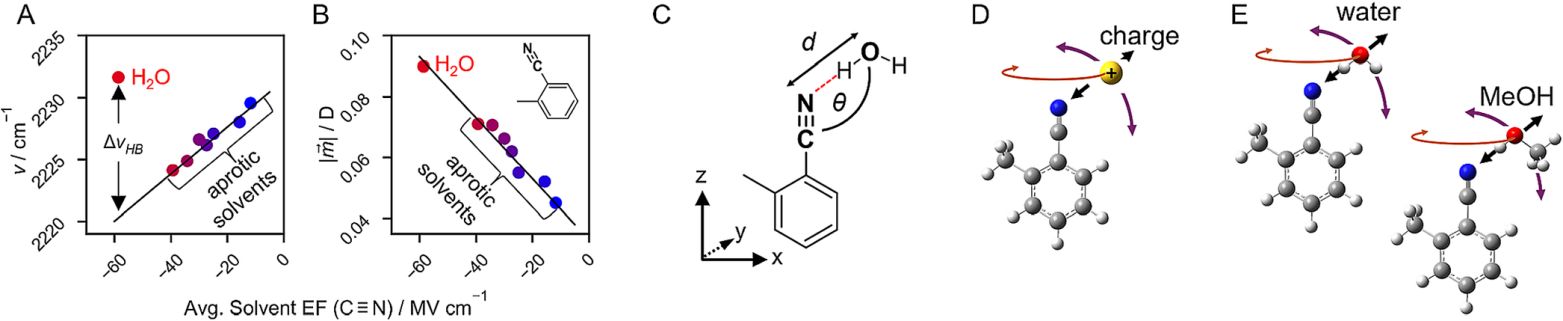
The C≡N stretch transition dipole moment (TDM)  is a linear electric field probe, in contrast to the vibrational frequency ν, which is complicated by the HB blueshift . (A) The experimental ν of the C≡N stretch of oTN (*o*-tolunitrile, see inset in B) shows a linear trend with electric field that can be modeled with the linear VSE eq 1b (black line) only for aprotic solvents. In water, a deviation from the line is observed, indicating C≡N frequencies require an additional term, , to account for H-bonding interactions (eq 2). (B) In contrast,  increases linearly with electric fields in aprotic solvents and water, as modeled with the VSE (eq 3a; black line). (C) We model  in terms of the heavy atom HB distance *d*(C≡N–O_HB-donor_) and the HB angle θ(C≡N–O_HB-donor_), *d* and θ, respectively. (D, E) To derive this model, density functional theory (DFT) calculations were performed to obtain nitrile frequencies and TDMs for oTN in two types of environments: (D) purely electrostatic environments where the nitrile interacts with a positive point charge and (E) H-bonding environments where the nitrile interacts with a water or methanol (MeOH) molecule; in all cases, the distance (black arrows) and angle (violet and orange arrows) of the interacting particle were varied. A and B are reproduced with permission from ref (50). Copyright 2022 American Chemical Society.

The nitrile (C≡N) stretch is the most commonly used vibrational probe,^14,37−44^ since it appears in an uncluttered region of the infrared (IR) spectrum and because nitriles are easily introduced into biological environments like proteins (via drugs or noncanonical amino acids)^39,45,46^ or chemical settings like surfaces.^19,20,47^ Despite its popularity, C≡N frequency tuning can exhibit complicated behavior that does not always follow the VSE (Figure 1A). In aprotic environments, the C≡N stretch shows a linear -behavior as described by eq 1b. However, in H-bonding environments, anomalous frequency shifts are observed which are inconsistent with eq 1b.^18,19,38,48−50^ Further, this anomalous behavior cannot be explained by relevant quadratic electric field contributions due to Δα, that is, eq 1a also cannot describe the frequency tuning.^50−52^ Instead, a description of nitrile frequencies requires the introduction of an additional variable called the HB blueshift ,^48,53,54^ to account for ‘C≡N···H’ interactions:
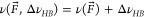
2where  is defined in eq 1a/1b.

Various approaches have attempted to indirectly correct for this anomaly in the nitrile’s -behavior in H-bonding environments via temperature-dependent experiments,^54^ correlations with nuclear magnetic resonance,^48^ or molecular dynamics (MD).^40^ Recently, we found a new, direct approach to circumvent the issues with nitrile frequencies when we observed that the integrated IR absorption intensity (*I*_*IR*_) of nitriles varies monotonically with the electric field in both aprotic and protic solvents (Figure 1B).^50^ This additional VSE is explained by the dependence of the transition dipole moment (TDM; ), which governs the IR absorbance, with the electric field according to
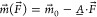
3awith
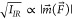
3bwhere  and  are the zero-field transition dipole and the transition dipole polarizability, respectively.^50^ Importantly, measuring nitrile TDMs enables quantification of nitrile electric fields in H-bonding environments by using eq 3a.^50,52^ In addition, jointly interpreting the nitrile’s TDM and frequency using eq 3a and eq 2, respectively, enables quantification of the anomalous HB blueshift .^50^

In our recent study, we measured nitrile frequencies and TDMs to *directly* assess nitrile HB blueshifts for the first time.^50^ The new TDM-based method showed that  can adopt values in a large range from 2 to 22 cm^–1^ in distinct solvent or protein environments.^48,50,53^ Consequently, we wondered whether the blueshift’s magnitude could be a useful metric to describe H-bonding, that is, if  in eq 2 could be mathematically modeled. Previous theoretical work explored the complicated vibrational behavior of the C≡N group and suggested that the anomalous -trend stems from nonnegligible higher order multipole effects^34^ or from contributions due to Pauli repulsion.^38^ Further, previous work^34,50^ implied that  may be a HB angle-dependent term (Figure 1C), which would be consistent with both proposed physical origins. The lack of intuition for the blueshift’s magnitude motivates the need to model  in a physically interpretable form.

Herein, we systematically explore HB blueshifts of the C≡N probe with the aim to find a simple, analytical expression for this observable. Toward this goal, we combined results from density functional theory (DFT)^55^ and the AMOEBA polarizable force field^56^ to generate a calibration for the vibrational response of the nitrile-containing molecule *o*-tolunitrile (oTN; see Figure 1C). In this approach, DFT was used to obtain C≡N vibrational frequencies and TDMs in a large set of purely electrostatic and H-bonding environments (∼1000 geometric configurations) including point charges (Figure 1D) and water and methanol (MeOH) molecules (Figure 1E), respectively. Then, the corresponding electric fields exerted on the C≡N were derived from the AMOEBA force field. We attempted to recapitulate the DFT-based frequencies using the VSE (eq 1a), which was (expectedly) unsuccessful due to the HB blueshift; in contrast, DFT TDMs are well-described by their corresponding VSE equation (eq 3a), highlighting the different frequency/TDM behaviors that were experimentally observed (Figure 1A, B).^50^ We modeled the DFT-derived HB blueshift as a function of HB distance and angle and successfully formulated a quantitative “HB blueshift-vs-HB geometry” relationship. We demonstrate the applicability of this relationship by comparison with experimentally derived blueshifts: we find that  for nitriles with rigid HBs can directly report on HB geometries, while  values for nitriles with fluctuating HBs are approximately halved from values predicted using geometry due to motional narrowing.

## Results and Discussion

### Modeling DFT-Based Frequencies and Transition Dipole Moments Using the Vibrational Stark Effect

In order to find an empirical relation for the HB shift, we chose a DFT-based strategy in which individual positive point charges (125 cases; Figure 1D) or individual water or methanol molecules (420 cases each; Figure 1E) were placed around oTN’s C≡N to model attractive purely electrostatic interactions or H-bonding interactions, respectively. These poses were optimized and normal mode analysis was performed to extract nitrile frequencies and TDMs (b3lyp/6-311++g** level of theory^57−61^ with GD3 dispersion correction^62^; see SI for the Methods Section with further references^83−90^). oTN was chosen as our model molecule because it is the side chain fragment of the nonnatural amino acid *o*-cyanophenylalanine (oCNF), with which we previously developed and applied the new TDM-based analysis in solvent and protein environments.^50,52^ The charges and molecules were positioned at N_C≡N_–charge or N_C≡N_–O_HB donor_ distances (*d*), respectively, ranging from 5.0–8.0 Å for point charges and 2.5–5.0 Å for HB donors, and C≡N–charge and C≡N–O_HB donor_ angles (θ) of 70–175° were used (Figure 1C). The HB distance range was motivated by typical radial distribution functions of HBs, which have a first solvation sphere centered around 2.5–3.5 Å.^63^ The angle range encapsulates HBs which vary from head-on (∼180°) to side-on (∼90°). Note that the ideal head-on angle of 180° was not used due to convergence issues in the DFT calculations. The DFT-derived vibrational frequencies (ν) and TDM magnitudes () were scaled by 0.9598^64^ and 0.4464, respectively, to match the experimental zero-field observables^50^ (see Methods Section).

**Figure 2:**
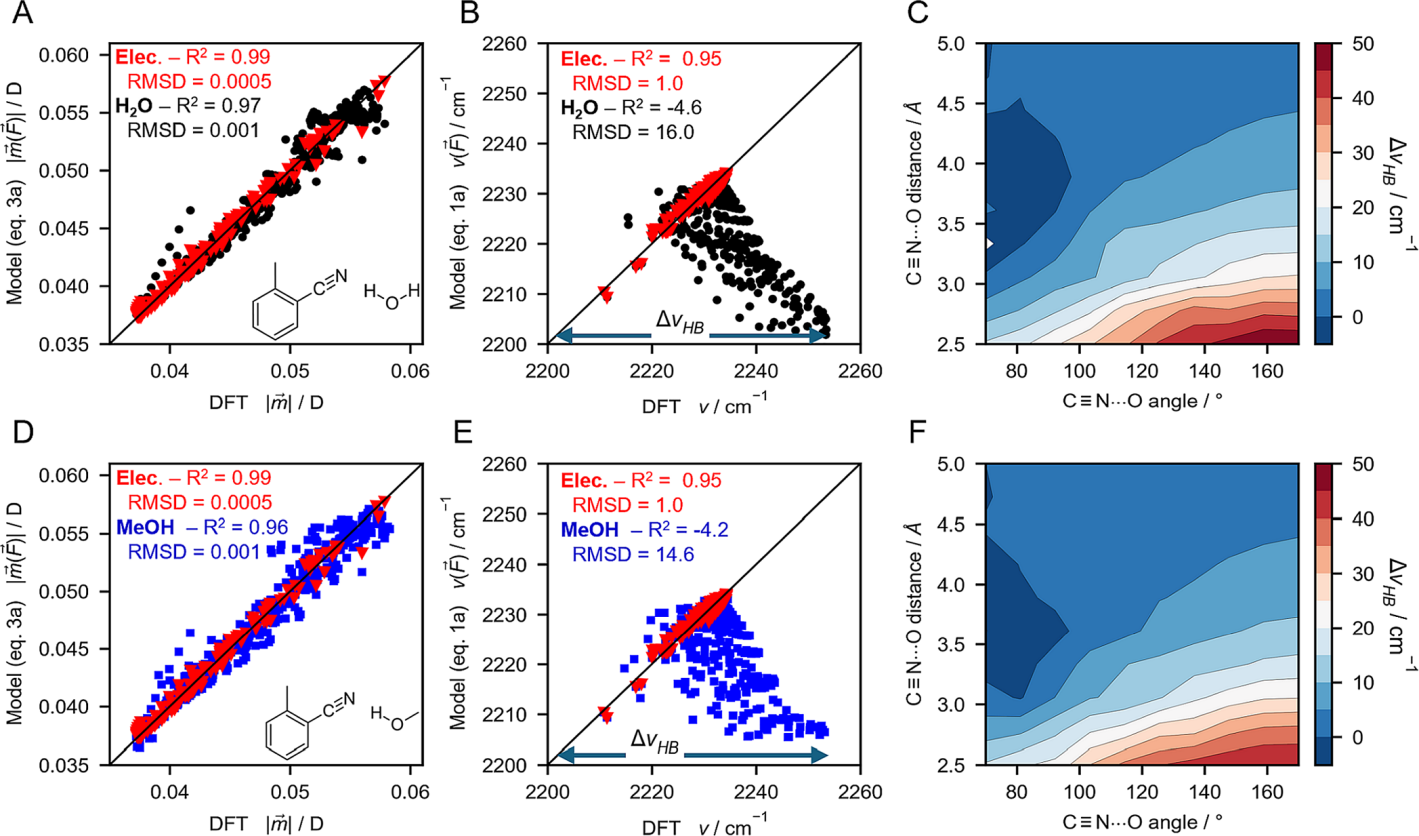
VSE modeling of DFT-based TDMs and vibrational frequencies (eqs 3a and 1a, respectively; exact analytical forms are shown in eqs S1 and S2) for oTN’s C≡N stretching mode in purely electrostatic environments with point charges and in H-bonding environments with water (A, B, C) and methanol (D, E, F). A, D: Correlation plots between modeled and DFT-based TDMs demonstrate eq 3a accurately describes nitrile environments with purely electrostatic perturbations (red triangles in A and D), water as a HB donor (black circles in A), and methanol as a HB donor (blue squares in D). Fitting parameters for all three environments are reported in Table S1. B, E: Correlation plots between modeled and DFT-based vibrational frequencies indicate that eq 1a only applies to nitriles under purely electrostatic perturbations (red triangles in B and E; fitting parameters reported in Table S2); when water or methanol are HB donors (black circles in B and blue squares in E, respectively), no correlation (R^2^ < 0) is found. The HB blueshift, , is determined as the difference between the DFT-predicted frequency and the black line representing ideal correlation and is illustrated by the double headed horizontal arrows. C, F: 2D heat plots of  dependence on heavy atom HB distance and angle [*d*(C≡N–O_water/MeOH_) and θ(C≡N–O_water/MeOH_)].

Using DFT, we obtained  and ν values for oTN of 0.037–0.060 D and 2210–2255 cm^–1^, respectively (see *x*-axes in Figure 2A, B), which are consistent with prior experimental observations for aromatic nitriles (see Figure 1A, B).^18,25,40,50,52,53^ From the observed ranges it can be seen that purely electrostatic and H-bonding environments give rise to similar values for  (Figure 2A), consistent with eq 3a’s indication that  is only a function of . For the frequencies (Figure 2B), purely electrostatic perturbations produce ν values below the gas phase frequency of 2232 cm^–1^, consistent with attractive electrostatic C≡N–charge interactions. In contrast, most H-bonding environments with water give rise to frequencies >2232 cm^–1^, indicative of the HB blueshift.

To further underscore the difference in behavior exhibited by the frequencies vs the TDMs, we used the corresponding VSE equations including quadratic electric field contributions (eq 1a and extension of eq 3a; see eq S2 and eq S1, respectively) to model the DFT-based vibrational observables solely as functions of . Toward this goal, we used the polarizable AMOEBA force field^56^ to extract the electric field vectors () along the C≡N group for the DFT-optimized structures (see Methods Section for further details). AMOEBA-based electric fields rather than DFT-based electric fields were used for our analysis since the VSE parameters obtained herein enable prediction of vibrational spectra from AMOEBA MD simulations (see below; additionally, see SI Section 2 for further discussion on determination of electric fields from MD vs DFT); moreover, previous work showed that electric fields from AMOEBA agree remarkably well with experimental assessments.^52,65^ All VSE parameters were allowed to freely vary when fitting the VSE equations against the DFT results (see Tables S1 and S2). For the TDMs, we found that the VSE modeled the DFT results for purely electrostatic and H-bonding perturbations very well with R^2^ > 0.97 (Figure 2A). This is consistent with our previous experimental results that TDMs give direct access to the local nitrile electric field in both non-H-bonding and H-bonding environments (Figure 1A).^50,52^ Further, this modeling provides a good estimate of the experimentally derived linear field sensitivity of  (as discussed in SI Section 3; values are “experimentally derived” since they combine experimental spectra and MD simulations).^50^

In contrast to the TDMs, the C≡N vibrational frequency shifts are modeled well with eq 1a for purely electrostatic perturbations but extremely poorly for nitriles with HBs to water molecules (Figure 2B). For purely electrostatic perturbations, the correlation between the modeled and DFT frequencies is very good with R^2^ of 0.95. This modeling resulted in a Stark tuning rate of  (Table S2), which is impressively close to the experimentally derived value of .^50^ However, when H-bonded data points are modeled with eq 1a using the same parameters, an extremely poor correlation of R^2^ = −4.6 is obtained, implying that eq 1a provides a worse description than just modeling the data with its mean value. The bulk of the deviating data points are located below the line of perfect correlation, i.e., the DFT frequencies are larger than those predicted using eq 1a. We interpret the magnitude of this deviation (along the *x*-axis in Figure 2B) as the HB blueshift  (eq 2; see Figure 1A).

To verify that this behavior is not specific to water, we used methanol as an alternative HB donor; this is an important test, as methanol is a model for the amino acid side chains of serine or threonine, and the largest experimentally observed  occurred for a threonine–nitrile interaction.^50^ We found that the VSE model’s ability to recapitulate the DFT results for TDMs is just as robust as in the case where water is the HB donor (R^2^ = 0.96, Figure 2D), and highly similar VSE parameters were obtained compared to those derived for water H-bonding scenarios (Table S1). Further, the correlation of VSE (eq 1a) and DFT ν values for nitriles with methanol HBs is just as poor as the case for water HBs (R^2^ = −4.2, Figure 2E), confirming the blueshift behavior is not specific to water.

### Modeling the HB Blueshift as a HB-Geometry-Dependent Observable

In order to understand the unilateral deviation of the ν values modeled with eq 1a compared to the DFT frequencies in H-bonding conditions, we hypothesized that  is a HB-geometry-dependent value, i.e., it depends on the HB-heavy atom distance *d*(C≡N–O_water/MeOH_) and the HB-heavy atom angle θ(C≡N–O_water/MeOH_) (*d* and θ in Figure 1C). Note that we chose heavy atom-based distances and angles instead of the C≡N–H_water/MeOH_ geometry used in other work^54,66^ due to inaccuracies in hydrogen atom positions in MD simulations introduced by frequently used constraint algorithms;^67^ furthermore, a calibration with heavy-atoms enables comparisons with protein crystal structures, where protons are rarely resolved. Extracting the  values from Figure 2B,E and the corresponding *d*(C≡N–O_water/MeOH_) and θ(C≡N–O_water/MeOH_) from the DFT-optimized geometries, we can visualize the  geometry dependences for water and methanol HBs as 2D heat plots in Figure 2C and Figure 2F, respectively. In both cases, we observe two trends: in going from short (2.5 Å) to long (5.0 Å) distances,  decreases steadily toward zero, with slightly negative values at intermediate distances (3.5–4.0 Å) for side-on HBs (see transition from blue to dark blue to blue at angles of 70–90° and distances of 3.0–4.5 Å; Figure 2C, F); at the same time,  decreases while going from head-on (175°) to side-on HBs (70°).

**Figure 3:**
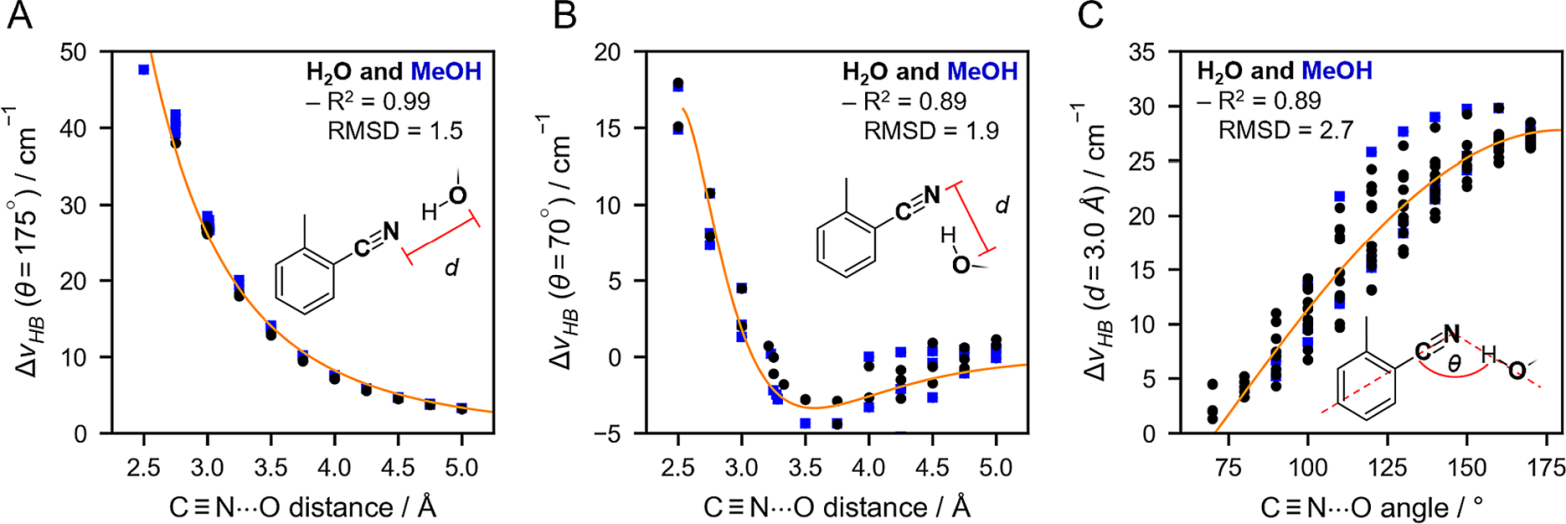
Models for  dependence on HB distance and angle based on data from Figure 2C,F (black and blue data points for water and methanol as the HB donor to the nitrile, respectively). A: HB distance dependence of  for head-on HBs (θ(C≡N–O_water/MeOH_) = 175°) can be modeled with an asymptotic form , with  (solid line; R^2^ = 0.99 and RMSD = 1.5 cm^–1^) or an exponential function , with *a* = 1.30 (dashed line; R^2^ = 0.99 and RMSD = 1.3 cm^–1^). B: HB distance dependence of  for side-on HBs (θ(C≡N–O_water/MeOH_) = 70°) is modeled with a Buckingham-like function , with , , and  (R^2^ = 0.89 and RMSD = 1.9 cm^–1^). Note that the Buckingham potential becomes unphysical at distances <2.5 Å and therefore is not shown. C: HB angle dependence of  at a constant HB distance *d*(C≡N–O_water/MeOH_) = 3.0 Å can be modeled using  with *m* = 0.82 (R^2^ = 0.89 and RMSD = 2.7 cm^–1^). See Table S3 for the complete list of optimized parameters.

By extracting the  values for head-on or side-on HBs, we can quantify the distance-dependence of . We combined the data sets for water and methanol HBs, and we found that head-on HBs (θ = 175°) demonstrate an asymptotic trend (Figure 3A) which decays from ∼50 cm^–1^ at 2.5 Å to ∼5 cm^–1^ at 5.0 Å according to a power law
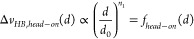
4awith *n*_1_ ≈ −4.0 or to an exponential function
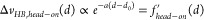
4bwith a decay constant of *a* ≈ 1.3 Å^–1^ (both with R^2^ = 0.99). While eq 4a is reminiscent of the energetic contribution from (repulsive) dipole–quadrupole interactions, which have a *d*^–4^ distance dependence,^68^eq 4b suggests contributions due to Pauli repulsion.^69^ To test if higher multipole interactions can have repulsive contributions leading to HB blueshifts, we show an energy decomposition for a C≡N···H_2_O HB in SI Section 4. Indeed, higher multipoles included in the AMOEBA force field, which describe interactions beyond the C≡N dipole-electric field interaction, show positive energetic contributions in support of eq 4a. In contrast, even though Pauli repulsion effects would generally be in line with blueshifts,^38^ the exponential decay constant is inconsistent with values used in molecular solids force fields to model it (2.7–4.6 Å^–1^).^70^ Instead, the *a* ≈ 1.3 Å^–1^ decay constant suggests a longer range interaction than would be expected for Pauli repulsion. The case against Pauli repulsion is further made by DFT frequency calculations for short-range, head-on C≡N interactions with an “electrostatically passive” Ne atom (i.e., no charge, dipole, quadrupole, etc.) where van der Waals interactions and Pauli repulsion should dominate the nitrile frequency tuning: in these calculations, *redshifts* are observed rather than blueshifts (reported in SI Section 4). As such, our results are in line with a multipole-based interpretation of the blueshift’s origin, while noting that a convolution with Pauli repulsion cannot be definitively ruled out. Importantly, our interpretation of the distance dependences is consistent with previous explanations of  describing other interaction types not included in the dipolar VSE eqs (eqs 1a/1b).^34^

When evaluating side-on HBs (θ = 70°), we note a more complicated asymptotic distance dependence with a minimum at roughly 3.5 Å (as noted above), at which point  is about −5 cm^–1^; this is followed by a gradual increase of  at larger *d*, becoming nearly negligible around 5.0 Å. We modeled this distance dependence with a Buckingham-like function (R^2^ = 0.89),^71,72^
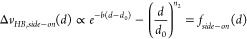
5which described the well at ∼3.5 Å more accurately than Lennard-Jones, Morse, or buffered 7–14 shapes (see Figure S4). We extracted values for the exponential decay constant (*b*) and the exponent of the power-term (*n*_2_) of *b* ≈ 3.1 Å^–1^ and *n*_2_ ≈ −8.2. In this case, the exponential decay constant is similar to values used in force fields for modeling Pauli repulsion,^70^ suggesting that HB blueshifts in side-on HBs originate from it; this finding is consistent with previous studies^38^ (and with blueshifts in DFT due to side-on interactions with a Ne atom, see SI Section 4). The power law in the Buckingham potential is typically used with an exponent of −6 to account for attractive dipole–induced dipole interactions. However, the original form of the Buckingham potential also included a *d*^–8^ term accounting for attractive quadrupole–induced quadrupole interactions.^71,72^ When fitting the data in Figure 3B using two power-terms, both exponents converged to the same value of ∼−8.2, indicating this value is fairly robust; as such, we tentatively assign negative contributions to  (i.e., redshifts) to induced higher-order multipole interactions.

In a similar fashion, we extracted the angular dependence of  at a HB distance of 3.0 Å, the average HB distance found in solvents and proteins (Figures S8 and S9; Table S8) and the distance where the side-on HB effect should be close to negligible (see Figure 3B). We used the relation
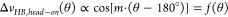
6to model the data points, and the best fit yielded *m* = 0.82 (R^2^ = 0.89), which accounts for the zero crossing at ∼70° (Figure 3B) by altering the cosine period. This deviation from *m* = 1 can be understood when taking into account that a side-on HB interacting with the π-orbitals of the C≡N would occur at θ(C≡N–O_water/MeOH_) ≈ 70–80° (Figure S5), and this is the point at which the cosine function should be 0.

**Figure 4:**
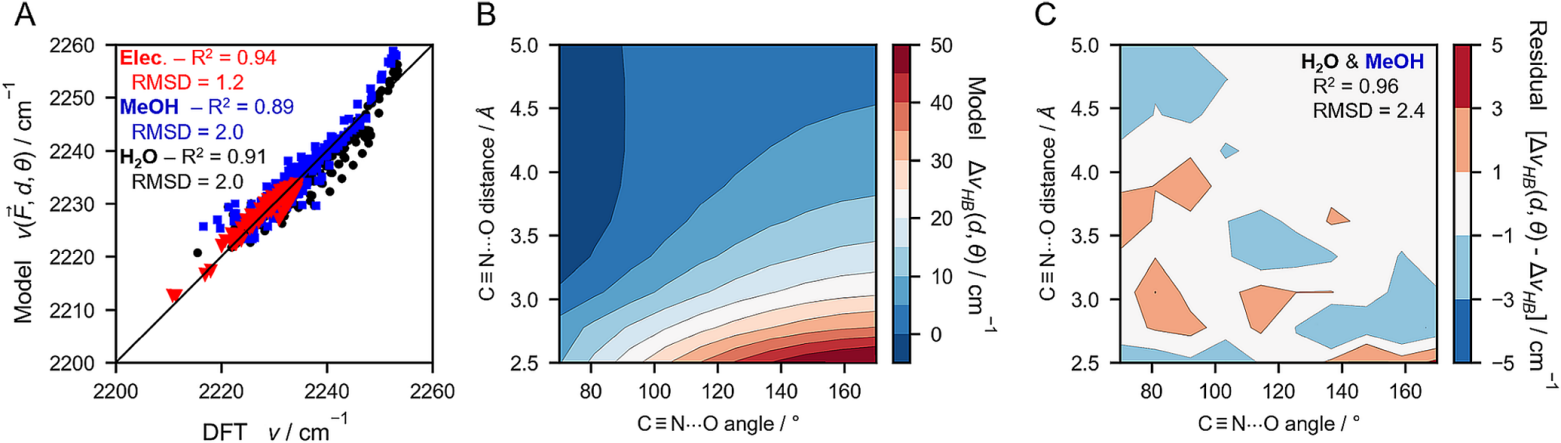
DFT-based vibrational frequencies for oTN’s C≡N stretching mode are recapitulated well when modeled as a function of electric field, HB distance, and HB angle. A: Correlation plot of  modeled (eq 2 utilizing eqs 1a and 7) and DFT-based vibrational frequencies shows that the model works well both for purely electrostatic perturbations (red triangles) and in the presence of water and methanol HBs (black circles and blue squares, respectively) with an overall R^2^ = 0.92 and RMSD = 1.8 cm^–1^ (R^2^ and RMSD values for each distinct environment are shown in the graph). Fitting parameters for the model are reported in Table 1 and Table S4. B: 2D heat plot of  with water and methanol as HB donors according to the model in eq 7. C: 2D heat plot of the residuals between modeled  (see B) and  from Figure 2C,F (R^2^ = 0.96 and RMSD = 2.4 cm^–1^).

Combining these dependencies, we now propose a HB-geometry-dependent relation for  composed of eqs 4a, 5, and 6 with the exponents set to integer values *n*_1_ = −4 and *n*_2_ = −8:
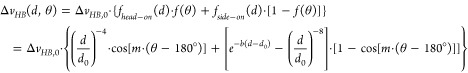
7(see SI Section 3 for the alternative form using the exponential function eq 4b for head-on HBs). Here,  is the HB blueshift at a reference distance *d*_0_ chosen as the point at which the Buckingham shape crosses zero. Further,  is the angular term in eq 6 which modulates the contributions of the head-on and side-on distance dependences of eqs 4a and 5, respectively. We modeled the C≡N frequency for nitriles experiencing purely electrostatic perturbations, HBs with water, and HBs with methanol simultaneously as a function of electric field, HB distance, and HB angle, i.e., using eq 2 with eq 7 for the  term. The resulting “ model vs DFT” plot (Figure 4A) shows that the VSE (eq 1a) with the addition of eq 7 recapitulates the DFT frequencies for purely electrostatic environments just as well as the VSE model alone (Figure 2B, E) but significantly improves the recapitulatability in H-bonding environments. Specifically, the fitting quality was effectively unaltered for purely electrostatic perturbations (from R^2^ = 0.95 to 0.94) but drastically improved in H-bonding environments (from R^2^ < 0 to ∼0.9). In this fit, the previously optimized VSE and empirical H-bonding parameters remain similar to those obtained in Figure 2 and Figure 3 with , a side-on exponential decay constant of *b* = 2.85 Å^–1^, and a cosine period modulation of *m* = 0.91. When we visualized the dependence of (*d, θ*) in eq 7 on *d*(C≡N–O_water/MeOH_) and θ(C≡N–O_water/MeOH_) as a 2D heat plot (Figure 4B), we found a highly analogous profile to those in Figure 2C and Figure 2F with a similarly broad range of values adopted (−5 to 50 cm^–1^), showing that eq 7 can recapitulate the DFT HB blueshifts with high accuracy. A 2D heat plot of the residuals between  (eq 7) and  obtained from DFT (Figure 2C, F) has residual values ranging from just −3 to +3 cm^−1^ (Figure 4C; R^2^ = 0.96), further indicating eq 7 accurately describes the blueshift for many HB distance and angle combinations. Some of the largest residuals are found for angles corresponding with side-on HBs, where the Buckingham potential slightly underestimates (Figure 3B). Even though eq 7 takes the form of a lengthy expression, only four parameters are needed to sufficiently tune the distance and angle dependence (Table 1), and all of them carry physical meaning in terms of describing specific underlying intermolecular interactions.

**Table 1:** Optimized Parameters for the HB Geometry-Dependent Model in eq 7 Describing the HB Blueshift in Figure 4B^a^

Parameters	Optimized values
*d*_0_/Å	3.36 ± 0.03
	16.6 ± 0.6
*b*/Å^–1^	2.85 ± 0.14
*m*	0.91 ± 0.01

a The corresponding VSE parameters are provided in Table S4.

As shown in SI Section 8, we narrowed down Figure 4B to a relevant regime of commonly adopted HB geometries in solvents. Based on AMOEBA MD simulations of oTN in water and methanol (see details in SI Section 1), the average HB distance decreases monotonically from 3.35 Å for side-on HBs (70°) to 2.93 Å when head-on HBs are adopted. Our model (eq 7) predicts  for side-on HBs interacting with the C≡N’s π-orbitals [∼70° for θ(C≡N–O)]. As the angle and distance concomitantly increase and decrease, respectively, the blueshift increases steadily, plateauing around 26 cm^–1^ for head-on HBs with θ(C≡N–O) >170°. Furthermore, we also investigated the HB blueshift in the (rare) case of two simultaneous HBs with a nitrile by comparing DFT blueshifts with values derived using eq 7 (SI Section 7): we found that summing  for each HB was an accurate model, implying each H-bonding interaction can be treated independently.

### Testing the HB-Geometry-Dependent Model for the HB Blueshift against Experimental Data: Rigid HBs

Based on the HB geometry-dependent model’s ability to recapitulate the nitrile DFT frequencies, we sought to test the model by comparing predicted blueshifts against experimental data for cases with nitriles in H-bonded environments. Toward this goal, we revisited our recent work, in which we introduced the noncanonical amino acid oCNF into photoactive yellow protein (PYP).^50,52^ In this previous work, oCNF was incorporated into PYP in place of endogenous phenylalanines (F), resulting in two PYP variants, F92oCNF and F28oCNF, which were H-bonding and showed distinct  values with moderate to large magnitudes. In the following, we reanalyze our previously obtained data (namely, IR spectra, crystal structures, and MD simulations) to enable comparisons between experimentally derived HB blueshifts () and HB blueshifts predicted from MD simulations using eq 7 (i.e., ).^50^

**Figure 5:**
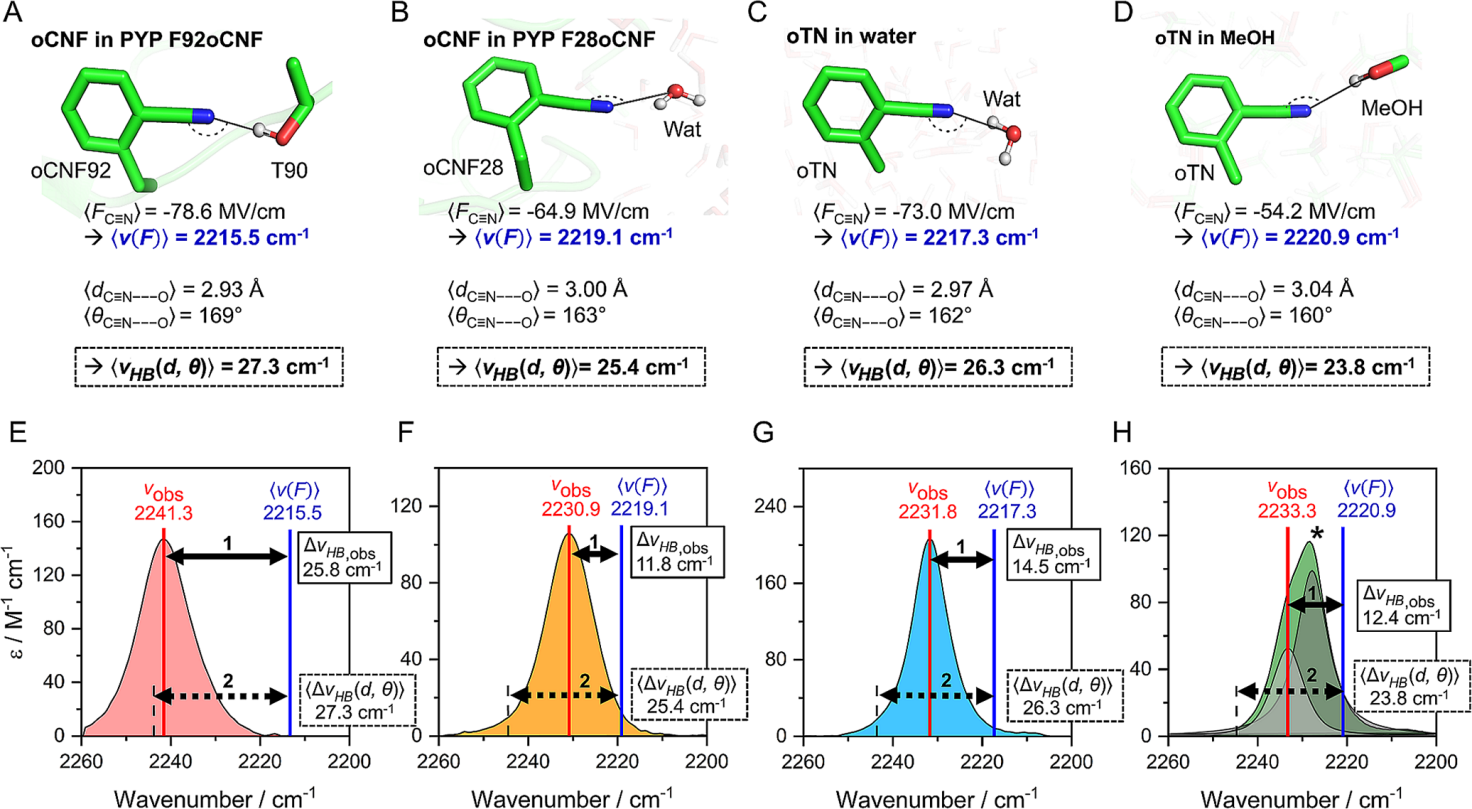
Testing the HB geometry-dependent model  against experimentally observed blueshifts for nitriles in proteins and solvents. A, B, C, D: Representative C≡N H-bonding AMOEBA MD structures of noncanonical amino acid oCNF in PYP variants F92oCNF (A) and F28oCNF (B) and of oTN in water (C) and methanol (D). Average electric fields on the C≡N in H-bonding fractions from MD simulations are shown below the structures,^52^ and the resulting VSE-based frequencies  (eq 1b) are shown highlighted in blue. Further below, average HB donor distances and angles from the simulations^52^ are shown together with the resulting average HB blueshift  (eq 7) in a dashed-line box. E, F, G, H: Experimental determination of  using IR spectra of F92oCNF (E), F28oCNF (F), oTN in water (G), and oTN in methanol (H). The peak position is specified by a solid red line labeled with . C≡N frequencies due to the VSE alone are indicated by a blue vertical line labeled with  (see also A–D). The difference between  and , i.e., between the red and blue lines, is indicated by a solid double headed arrow (1) representing the experimentally observed HB blueshift . The dashed double headed arrow (2) represents the predicted HB blueshift  from A–D. For oTN in methanol (H), only the H-bonding fraction is evaluated (the non-H-bonding band is indicated by an asterisk; see SI Section 11 for more details). Data in E–G are reproduced with permission from ref (50). Copyright 2022 American Chemical Society.

Starting with F92oCNF (Figure 5A), X-ray crystallography showed that the C≡N group is engaged in a head-on HB with the hydroxyl group of threonine 90 (T90), and 100 ns long AMOEBA MD simulations indicated an average C≡N–HO-T90 HB distance and angle of 2.93 Å and 169°, respectively (see Figure 5A, a representative MD snapshot).^50,52^ Using the HB geometry-dependent model in eq 7, we derive an average predicted value of  = 27.3 cm^–1^, a large value as expected for a head-on HB (Figure 4B). To compare this value to experimental results (Figure 5E), we also determined the C≡N’s peak position due to the VSE alone (eq 1b); this was done by using the experimentally determined zero-field frequency and Stark tuning rate^50^ and the average electric field for the H-bonding fraction from MD (−78 MV/cm, ref (52); see SI Section 11 for further details). We obtain a VSE-based vibrational frequency of 2215.5 cm^–1^ (blue values in Figure 5A/Figure 5E and vertical blue line in Figure 5E). The experimental IR spectrum of F92oCNF has a peak position of 2241.3 cm^–1^,^50^ and subtracting the frequency for the VSE alone from the experimental frequency results in a HB blueshift of  = 25.8 cm^–1^ (eq 2). This experimentally derived blueshift matches very well with the HB geometry-based value of 27.2 cm^–1^, as indicated by the similar length of the solid and dashed double headed arrows in Figure 5E. We note that similar results are obtained when  is calculated from the distribution of  values obtained by applying eq 7 to each H-bonding frame of the MD simulation (see distributions for this and the following cases in Figure S15).

Further support for our HB geometry-based  model and the observation of large values for head-on and/or short HBs is provided by a new publication where a nitrile probe was incorporated into different metal organic frameworks (MOFs).^73^ The rigid MOF structure enabled the introduction of H-bonding moieties (allylic and aromatic carboxylic acids) near the nitrile. According to DFT, these C≡N–HO interactions occur at average HB distances/angles of 2.85 Å/168° (allylic acid; “AA”), 2.80 Å/148° (benzoic acid; “CPh”), and 2.79 Å/150° (isophthalic acid; “DCPh”). As in the case of F92oCNF,  and  are in excellent agreement: the experimental/predicted values (in cm^–1^) for AA are 36/31.8, for CPh are 29/28.2, and for DCPh are 33/32.2. It is interesting to note that the nitrile in the MOFs is an aliphatic C≡N, not an oTN derivative, and that the nitrile HB partners are carboxylic acids, not water or alcohols. These differences make the similarity between the experimental and predicted HB shifts all the more impressive; this comparison suggests that our model can work generally for H-bonded nitriles with different types of HB donors.

### Testing the HB-Geometry-Dependent Model for the HB Blueshift against Experimental Data: Fluctuating HBs

We next analyzed F28oCNF, where crystallography showed that the C≡N group is solvent exposed and H-bonded to bulk water;^50^ MD indicated this interaction has an average HB distance and angle of 3.00 Å and 163° (Figure 5B).^52^ Using eq 7, we obtained  = 25.4 cm^–1^ (Figure 5B). However, unlike F92oCNF, we noted a considerable discrepancy between this value and  when we analyzed F28oCNF’s IR spectra (Figure 5F). F28oCNF has an average electric field of −64.9 MV/cm in the MD H-bonding fraction (see ref (52)), and the pure VSE effect predicts the C≡N’s peak position to be at 2219.1 cm^–1^ (see values in Figure 5B and red line in Figure 5F). However, in the experimental IR spectrum, we observe a peak position at 2230.9 cm^–1^, which leads to  = 11.8 cm^–1^, only half as large as  (this is visually demonstrated in Figure 5F by the red line appearing halfway along the dashed double headed arrow). We hypothesized that this discrepancy may be related to F28oCNF’s H-bonding with the highly fluctuating solvent environment, in which the C≡N rapidly alternates between H-bonding and non-H-bonding states (see SI Section 10).

To test this idea, we analyzed the HB shifts for our model molecule oTN in water and methanol. From AMOEBA MD simulations, we find that oTN adopts similar HB geometries as F28oCNF, i.e., an average HB distance and angle of ∼3.0 Å and ∼160° with the nitrile (Figure 5C and D for water and methanol, respectively). Using eq 7, we obtain relatively large  values, with 26.3 cm^–1^ in water and 23.8 cm^–1^ in methanol (Figure 5C, D). Extracting  experimentally, we obtain  values of 15.2 and 13.1 cm^–1^ (Figure 5G and H, respectively), indicating  is much smaller than  like for F28oCNF. Note that in methanol (Figure 5H), H-bonded and non-H-bonded oTN populations are detected as two overlapping peaks (2233.3 and 2227.8 cm^–1^); we herein discuss only the H-bonded fraction (see SI Section 11 and Figure S13).

To reconcile the excellent match for F92oCNF and the MOFs but the disparity for F28oCNF and oTN in solvents, we must take into consideration the time scales under which HBs fluctuate for both groups. In F92oCNF (Figure 5A), the C≡N is engaged in an intraprotein HB: we detect extended periods of uninterrupted H-bonding and narrow HB distance and angle distributions in AMOEBA MD simulations (see SI Sections 9 and 10), indicating this HB experiences long residence times and minimal geometrical fluctuations. Because of this weakly fluctuating (rigid) C≡N–HO-T90 interaction, the  distribution (Figure 5A, E) directly reflects on the average HB geometry as derived from our model in eq 7 and Figure 4B. The same argument holds true for the MOFs, where the HB geometry is locked in place by the framework. These cases are classified as the *inhomogeneous limit* in IR spectroscopy,^74^ i.e. where IR spectra directly reflect the distribution of instantaneous vibrational frequencies. Instead, for F28oCNF and oTN in solvents, the H-bonding with bulk solvent is highly fluctuating, characterized in MD by short H-bonding residence times and broad HB distance/angle distributions (SI Sections 9 and 10). If these fluctuations are faster than the difference in the vibrational frequencies between the fluctuating substates (a vibrational frequency difference of ∼20 cm^–1^ corresponds to a time scale of ∼2 ps), the substates are not resolved in the IR spectrum but instead motionally narrowed toward one IR band with an averaged peak position^74^ (as occurs in coalescence in nuclear magnetic resonance);^75^ lifetimes for H-bonding and non-H-bonding nitrile states were extracted from MD simulations and qualitatively support this possibility (Figure S10 and Table S9).

**Figure 6:**
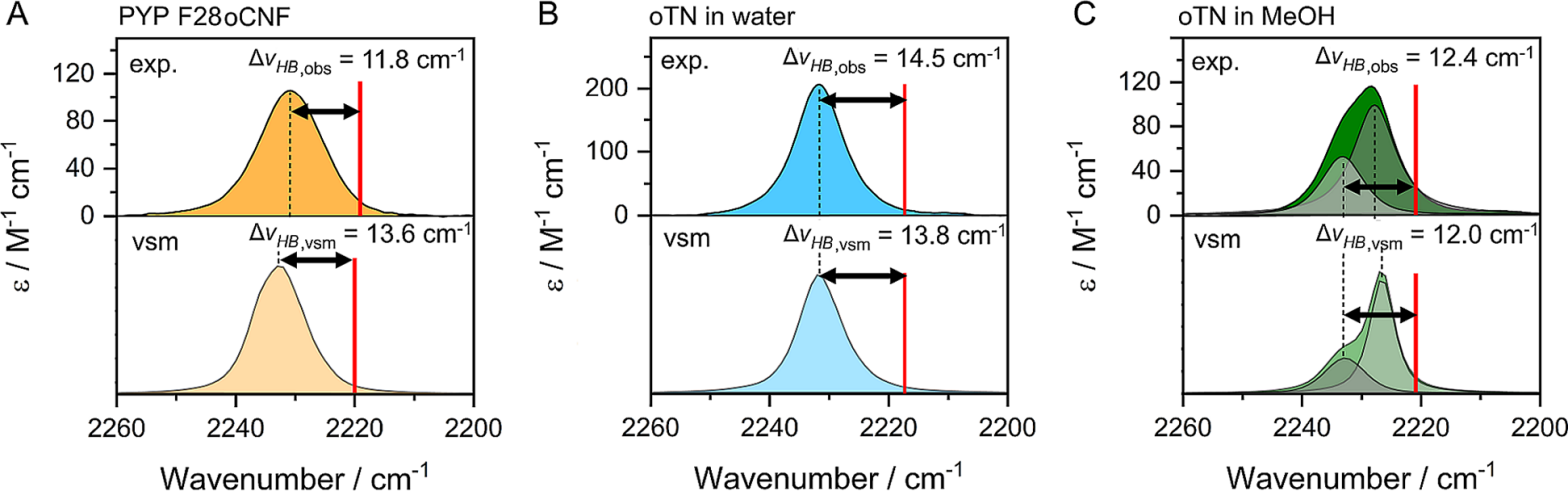
HB blueshifts for nitriles exposed to solvent environments are modulated by dynamics, i.e., HB fluctuations. A, B, C: Top row shows experimental IR spectra for the C≡N stretch in PYP F28oCNF (A), oTN in water (B) and oTN in methanol (C) as shown in Figure 5F, G, and H, respectively. The bottom row shows corresponding MD-based simulated IR spectra using eq 2 (with eqs 1a and 7) and parameters in Table 1 (and Table S4) as a vibrational spectroscopic map (vsm) obtained using the fluctuating frequency approximation (see Methods Section). The solid red vertical lines are predicted peak positions due to the VSE only (using eq 1b and parameters from ref (50)) in Figure 5F, G, and H, and the blueshifts derived from the vsm () are the difference between the simulated peak position and the frequency of the red line. Data in A and B is reproduced with permission from ref (50). Copyright 2022 American Chemical Society.

One way to test the hypothesis of motional narrowing is by applying IR lineshape theory.^74,76^ Accordingly, we used the parameters obtained from DFT to describe the C≡N TDM and frequency in terms of electrostatics and HB-geometry (Table 1 and Table S4; eqs 1a, 2, 3a, and 7) as a model to compute theoretical IR spectra from AMOEBA MD trajectories (referred to as a vibrational spectroscopic map, or “vsm”).^32^ Toward this goal, we first calculated the instantaneous C≡N TDMs and frequencies from MD simulations (performed every 20 fs over 2 ns in aggregate) for oTN in water and methanol and F28oCNF, and we utilized the well-documented fluctuating frequency approximation (FFA)^76,77^ to calculate MD-based IR spectra. In FFA, a Fourier transformation of the autocorrelation of transition dipole and frequency fluctuations is used to calculate realistic lineshapes (eq S3). Comparing the resulting computed IR spectra of oTN in water and methanol (Figure 6B and C, respectively) with those from experiment, we observe a very good recapitulation. In water, the simulated spectra yield one symmetric band for the C≡N stretch, with a peak position (2232 cm^–1^) almost identical to the experimental value; in methanol, the FFA-based spectra show an asymmetric line shape which occurs due to distinct H-bonded and non-H-bonded fractions absorbing at ∼2233 and ∼2228 cm^–1^, respectively, which are again quite similar for experimental and computed spectra. Importantly, we can take the difference between the vsm frequencies and the previously determined frequencies due to the VSE alone (Figure 5G, H; red lines in Figure 6B, C) to determine apparent  values of 14.5 and 12.7 cm^–1^ for water and methanol, respectively, which deviate from the experimentally obtained values by <0.7 cm^–1^, an impressively close match. We used the same approach to calculate the IR spectrum and vsm blueshift for F28oCNF (Figure 6A) and again obtain a good match for : comparing /, we observe values of 11.8/13.6 cm^–1^, i.e., a deviation of only 1.8 cm^–1^.

Overall, the vsm can recapitulate the experimental nitrile spectra with high accuracy. This demonstrates that our HB geometry-dependent model is not only robust in minimally fluctuating settings, but also in (quickly) fluctuating solvent or protein environments when dynamical effects are considered. More specifically, in the cases we tested with fluctuating HBs, the geometry dependent values of  = 23.8–26.3 cm^–1^ (Figure 5F–H) are reduced by a factor of roughly 2 to  12.0–13.8 cm^–1^ (Figure 6A–C). This reduction by a factor of 2 is what is expected for the simplest case when nitrile protic and aprotic subpopulations are interconverting with similarly fast exchange rates such that the geometry dependent value  will be averaged with 0 cm^–1^ (i.e., the blueshift for the non-H-bonded fraction). This exercise makes clear that knowledge of the dynamics experienced by a nitrile is key to prevent erroneous assessments of the HB geometry based on  alone: such dynamics can be evaluated using temperature dependent or two-dimensional IR experiments.^41,44,66,78^

**Figure 7:**
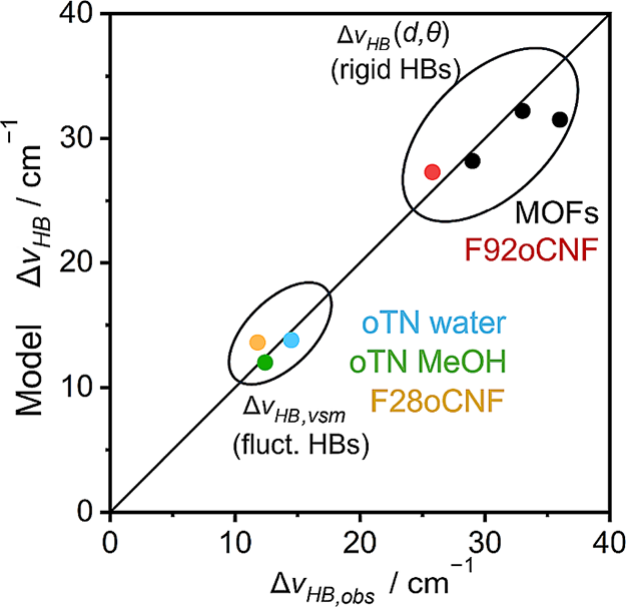
Experimentally derived HB blueshifts of nitriles can be recapitulated by accounting for HB geometry and dynamics. The plot shows a correlation between experimentally observed () and predicted () or simulated () blueshifts; when the appropriate model is used, the correlation is excellent (R^2^ = 0.95). The black diagonal is the perfect correlation with a slope of 1. Experimental values for the MOF data points are from ref (73).

To summarize the evaluation of our models for HB blueshifts, we correlated the experimental and predicted values for  in Figure 7. We find that calculating  from our HB geometry-dependent model in eq 7 works very well for rigid HBs in F92oCNF and MOFs, implying it is possible to extract information on HB geometry directly from HB blueshifts. For fluctuating HBs like F28oCNF and oTN in solvents, HB dynamics have to be considered, as described above: when they are, an excellent agreement between observed and modeled HB blueshifts is obtained (R^2^ = 0.95).

## Conclusions

Aiming to provide a simple, empirical description for the anomalous HB blueshift of nitriles, we developed a model that describes HB effects on C≡N frequencies as the sum of the widely used VSE and an additional term, . This model describes  in terms of HB geometry, i.e., HB heavy atom distance *d*(C≡N–Donor) and angle θ(C≡N–Donor), and provides a framework that can be used to assess the H-bonding geometry of a C≡N bond in a broad range of protic environments. The physical basis for the distance and angle dependence is a combination of repulsive quadrupolar electrostatic interactions, Pauli repulsion, and attractive multipolar interactions, supporting previous interpretations of the blueshift’s origin(s).^34,38^ These findings further expand on theoretical models that have aimed to understand H-bonding in terms of its quantum and/or classical mechanical nature, many of which have pointed toward a dominant (classical) electrostatic character.^79−81^ We found an important third contributor to , the HB dynamics, also needs to be considered when using the model developed herein.  values of rigid HBs with long residence times and minimal fluctuations are directly dependent on HB geometry; in contrast, nitrile IR bands for quickly fluctuating HBs experience motional narrowing, altering their lineshapes. Consequently, HB residence/exchange times should be considered when estimating HB geometry via . In closing, we emphasize that the nitrile blueshift model presented in eq 7 works well for MOFs which had a different type of nitrile and different HB donors. This suggests that the model developed here is broadly applicable and can be used to characterize HBs for nitriles on diverse substrates, ranging from drugs to amino acids, and in diverse settings, ranging from electrodes to microdroplets to proteins.^19,20,37,42,44,50,52,66,82^

## Data Availability

Data supporting the findings of this work are deposited under https://github.com/KozuchLab/Publications/tree/0b6decb2982a9f1624353bcf3bd8fb6c4f212dd6/CN_HB_blueshift or are available upon reasonable request from the authors.

## Supporting Information Available

Methods Section as well as further sections detailing and summarizing differences between DFT and AMOEBA-based electric fields, the fitting results from Figure 2, alternative models for Figure 3, DFT-based tests of multipolar and Pauli repulsion effects, explanation on the parameter *m* in eq 7, modeling of two simultaneous HBs, HB radial distribution function from MD simulations of oTN, HB geometry and fluctuation analysis for oTN (in water and MeOH) and oCNF (F28oCNF and F92oCNF variants of PYP), further explanations for the determination of HB blueshifts and the calculation of IR spectra from MD simulations, and HB blueshifts determined by applying eq 7 to each MD frame, additional references (PDF)
