# Supplementary material for: Hydrogen Bond Blueshifts in Nitrile Vibrational Spectra Are Dictated by Hydrogen Bond Geometry and Dynamics
Source: JACS Au. 2024 Dec 5;4(12):4844–55. doi: 10.1021/jacsau.4c00811 (PMC11672138; doi:10.1021/jacsau.4c00811)
Supplement: Supplementary file 1 — au4c00811_si_001.pdf [file au4c00811_si_001.pdf]

Supporting Information for

Hydrogen Bond Blueshifts in Nitrile Vibrational Spectra are Dictated by Hydrogen Bond Geometry and Dynamics

Jacob M. Kirsh[a] & Jacek Kozuch[b,c,*]

[a] Department of Chemistry, Stanford University, Stanford, California 94305-5012, United States
[b] Freie Universität Berlin, Physics Department, Experimental Molecular Biophysics, Arnimallee 14, 14195, Germany
[c] Freie Universität Berlin, SupraFAB Research Building, Altensteinstr. 23a, 14195 Berlin, Germany

*email: jacek.kozuch@fu-berlin.de

Contents

1. Methods Section 2
2. (Conceptual) differences between AMOEBA-based and DFT-based electric fields 4
3. Fitting results for data in Figures 2, 3, and 4 6
4. Testing multipolar or Pauli repulsion in head-on or side-on HBs 9
5. Alternative models to fit the distance dependence for side-on HBs 11
6. Parameter m in angular dependence of the HB blueshift 12
7. Modeling HB blueshifts of two simultaneous HBs 13
8. Angle dependence for common HB distances in solvents – radial distribution functions of ortho-tolunitrile 14
9. HB analysis of MD trajectories: oTN in water and MeOH and oCNF in PYP 15
10. HB analysis of MD trajectories: HB residence times 19
11. Determination of HB blueshifts from experimental spectra 22
12. Distributions of HB blueshifts from AMOEBA MD simulations 25
13. References 26

1. Methods Section

Density Functional Theory and Molecular Mechanics-Based Calibrations

DFT calculations were performed using Gaussian 16 Rev A.03[1] with the B3LYP functional,[2–4] the GD3 dispersion correction,[5] and 6-311++g(d,p) as basis set;[6,7] harmonic vibrational frequencies and transition dipole moments (TDMs) were calculated using normal mode analysis and scaled with 0.9598[8] and 0.4464, respectively; the latter was determined to match the experimental value in vacuo (zero-electric field conditions).[9] Note that no systematic evaluations for TDM scaling factors are available in the literature, to the best of our knowledge; one study reports a range of scaling factors from 0.3 – 1.8 for rovibrational spectra.[10] For the geometry optimization, *o*-tolunitrile (oTN) was placed such that the C≡N bond was oriented along the z-axis (main text Figure 1C) and the aromatic phenyl groups was in the xz-plane. Calculations were performed with point charges (+0.25, +0.5, +0.75, or +1.00 e) placed at distances of 5.0, 6.0, 7.0 or 8.0 Å from the N atom along the unit vectors (0,0,1), (0,1,0), (1,0,0), $(0,\sqrt{0.5},\sqrt{0.5})$, $(\sqrt{0.5},0,\sqrt{0.5})$, $(\sqrt{0.5},\sqrt{0.5},0)$, and $(\sqrt{0.\bar{3}},\sqrt{0.\bar{3}},\sqrt{0.\bar{3}})$; this resulted in AMOEBA-based electric field magnitudes of up to 60 MV/cm acting on the C≡N group along x, y or z directions. For calculations with water and methanol (MeOH) as hydrogen bond (HB) donors, the HB donor atoms were constrained at C≡N---$O_{water/MeOH}$ distances from 2.5 – 5.0 Å (0.25 Å steps), at C≡N---$O_{water/MeOH}$ angles from 70° – 175° (in 10° steps between 70° and 170°), and 0°, 45° and 90° dihedrals between the aromatic plane and the $O_{water/MeOH}$. In addition, calculations were run with unconstrained distances and dihedrals but constrained angles from 70° - 175°. Overall, this amounted to 125 purely electrostatic conditions, 420 water H-bonded conditions, and 420 methanol H-bonded conditions. The optimized geometries were translated to the Tinker[11] format to calculate AMOEBA[12] force field-based electric field vectors along the C≡N bond. Electric field vectors were obtained as the average between the C and N atoms; atomistic fields were calculated from the induced dipoles divided by their polarizability (as implemented in the AMOEBA force field[13]). Parameters for oTN were taken from previous studies,[9] and water and methanol parameters came from the original amoeba09 force field.[13] VSE-based transition dipole magnitude changes and vibrational frequency changes were modeled via

$$|\vec{m}| = |\vec{m}|_0 + \sum_i A_{ii} \cdot F_i + \sum_i \sum_j F_i \cdot B_{ij} \cdot F_j \qquad \textit{eq. S1}$$

(6 fitted parameters; A_{yy}, B_{xx}, B_{xy}, and B_{xz} were set to zero as they did not improve the fits in main text Figure 2A and Figure 2D further) and

$$\bar{\nu} = \bar{\nu}_0 + |\Delta\vec{\mu}| \cdot F_z - \frac{1}{2}\sum_i F_i \cdot \Delta\alpha_{ii} \cdot F_i \qquad \textit{eq. S2}$$

(5 fitted parameters) respectively, where *i* = x, y, z.

Molecular Dynamics Simulations and Simulation of IR Spectra

Molecular dynamics simulations using the AMOEBA force field[12] were conducted as reported in our previous studies[14–17] using Tinker9.[11,18] Parameters for water and methanol were taken from the amoeba09 force field;[13] parameters for oTN and the noncanonical amino acids oCNF and *p*-coumaric acid (i.e., PYP's chromophore) were taken from our previous work,[14] where they were generated using Poltype2.[19] For the protein MD runs, the amoebabio18 force field was used.[12] For simulations of the model molecule oTN, 50 x 50 x 50 Å^3 boxes (under periodic boundary conditions) were filled with water or MeOH molecules as solvent and minimized using steepest decent until all forces were below 1 kcal mol^{-1} Å^{-1} with electrostatics and van der Waals cutoffs of 7 and 9 Å,

respectively, and dipole convergence (mutual) set to 0.01 D. NVT and NPT equilibrations were conducted at 1 fs time steps for 500 ps with van der Waals cutoff increased to 12 Å, the RESPA integrator, the Bussi thermostat, and Monte-Carlo barostat (300 K and 1 bar). MD production runs were conducted with 1 fs time steps over 10 ns under NPT conditions and dipole convergence thresholds set to 0.0001 D. Overall, 10,000 equally spaced frames were saved every 1 ps. The induced dipoles on the C and N atoms of the C≡N bond and the H-bonding partner coordinates within 5.0 Å were saved for each frame. Induced dipoles were translated to atomistic electric field vectors, which were then averaged to obtain the electric field vectors along the C≡N bond. Each frame was then classified as H-bonding or non-H-bonding: a HB was identified if a HB donor heavy atom was within 4.0 Å of the N atom of the C≡N bond and if the N---donor-hydrogen angle was < 30° (HB cone). The coordinates of the C≡N bond and the HB donor heavy atom were used to determine HB distance and angle. Results from 100 ns AMOEBA MD simulations were taken from our previous work[14] and reanalyzed: these MD simulations were performed according to the same protocol, with the exception of using a larger simulation box of 75 x 75 x 75 $Å^3$ and addition of NaCl at a final concentration of 50 mM.

For the calculation of MD-based IR spectra based on the VSE and our HB-geometry dependent model used as a vibrational spectroscopic map (Figure 6 in the main text), we repeated the MD production runs of oTN in water, oTN in MeOH, F28oCNF PYP, and F92oCNF PYP. For each case, we performed ten 200 ps runs (a total of 2.0 ns) with outputs saved every 20 fs. Again, electric fields along the C≡N were determined for each frame and coordinates of HB partners were extracted within 5.0 Å; here, 5.0 Å was used as the cutoff distance since Figures 2, 3, and 4 indicated that $\Delta\bar{\nu}_{HB}$ is negligible at HB distances > 5.0 Å. For each frame, the instantaneous vibrational wavenumber and transition dipole of the C≡N was calculated using main text eqs. 1, 2, 3, and 7. The time series of the fluctuating observables were then used to calculate the IR lineshape using the fluctuating frequency approximation[20,21] according to

$$I(\Delta\bar{\nu}) \propto \int_{-\infty}^{\infty} dt\ e^{-i\Delta\omega t} \langle M(0) \cdot M(t) \cdot \exp\left[i \int_{0}^{t} dt' \Delta\omega(t')\right] \cdot e^{-\frac{t}{2T_1}} \rangle \qquad \textit{eq. S3}$$

where $\Delta\bar{\nu}$ is the wavenumber shift from the average, $M(0)$ and $M(t)$ are the instantaneous transition dipoles at time zero and t, $\Delta\omega$ is the instantaneous frequency shift from the average frequency, and T_1 is the vibrational dephasing time (T_1 = 1 ps was used herein).

Infrared Spectroscopy

IR spectra of *o*-tolunitrile (oTN, TCI America) in methanol (anhydrous, > 99.0% purity; SigmaAldrich) were recorded, as reported previously,[9] at 1 cm^{-1} spectral resolution using a Bruker Vertex70 with a liquid nitrogen cooled MCT detector. oTN was dissolved at a concentration of 10 mM in methanol (determined by weighted mass) and filled into a transmission cell with a 56 μm spacer. The spacer thickness in the assembled cell was verified by measuring the interference fringes (to ±0.2 μm) to present the absorbance axis in units of extinction coefficient according to Lambert-Beer's law. Baseline corrections were performed using a polynomial function (typically degree five or lower) and lineshapes were fit simultaneously to the spectrum and its second derivative to restrain the fitted peak positions (see SI Section 9 for an example). IR spectra of oTN in water, F92oCNF PYP, and F28oCNF PYP were reused from our previous work (shared by Prof. Steven Boxer, Stanford University, USA).[9]

2. (Conceptual) differences between AMOEBA-based and DFT-based electric fields

As in previous work, we determined electric fields acting along the C≡N group as the average of the fields acting on the C and N atoms using the AMOEBA force field. To enable this, we determined the electric fields on the C and N atom $\vec{F}_{C,AMOEBA}$ and $\vec{F}_{N,AMOEBA}$, respectively (from the induced dipoles) and calculated the average:[17,22–24]

$$\vec{F}_{C\equiv N,AMOEBA} = \frac{\vec{F}_{C,AMOEBA} + \vec{F}_{N,AMOEBA}}{2}. \qquad \textit{eq. S4}$$

An alternative to this is the determination of the atomistic electric fields from DFT (e.g. using the keywork "prop=efg" in Gaussian). This keyword provides the electrostatic potential and the electric fields at the location of each atom such that the electric field can be calculated in two ways. First, one can use the difference in potential at both atoms, $\varphi_{C,DFT}$ and $\varphi_{N,DFT}$, divided by the C≡N bond length, $d_{C\equiv N}$,[25] approximating the derivative of the potential:

$$F_{C\equiv N,DFTesp} = -\frac{\varphi_{C,DFT} - \varphi_{N,DFT}}{d_{C\equiv N}}. \qquad \textit{eq. S5}$$

Alternatively, the same approach as in eq. S4 can be used, i.e. using the fields on each atom, $\vec{F}_{C,DFT}$ and $\vec{F}_{N,DFT}$:

$$\vec{F}_{C\equiv N,DFT} = \frac{\vec{F}_{C,DFT}+\vec{F}_{N,DFT}}{2}. \qquad \textit{eq. S6}$$

It is important to note that such atomistic electric fields from AMOEBA or DFT presented in eqs. S4 – S6 are not (necessarily) equivalent:

a) First, it is obvious that $F_{C\equiv N,DFTesp}$ is the magnitude of the electric field along the bond axis; instead $\vec{F}_{C\equiv N,AMOEBA}$ and $\vec{F}_{C\equiv N,DFT}$ are vectors. As such, information on off-axis contributions is lost when calculating $F_{C\equiv N,DFTesp}$.

b) Second, in classical force fields, the entire charge distribution is collapsed (electron density and nuclear charge) to atomistic parameters, such as an atomistic partial charge (and dipole, quadrupole, and polarizability in AMOEBA). In turn, in DFT programs like Gaussian, the electric field or potential is typically determined at the nucleus only, since it is not straightforward to determine the electronic charge density belonging to the atom under consideration. Therefore, MD-based fields refer to the force acting on the "entire" atom (nucleus + electrons) and due to polarization by the rest of the molecule. Instead, electric fields and potentials from DFT approaches contain polarization of the electron cloud of the same atom. We show in the following a comparison of the three methods relating to eqs. S4 – S6 using the example of oTN in the presence of a classical charge or a water molecule (Figure S1). For the sake of simplicity, we only discuss the electric field along the C≡N bond where the charge/water are positioned head-on to the nitrile.

According to Coulomb's law, we expect a field of ~ -45 MV/cm (negative signs indicated stabilizing interactions) at the C≡N group of oTN in the presence of a classical charge for the configuration shown in Figure S1 (left panel). AMOEBA provides a field in a similar range ($|\vec{F}_{C\equiv N,AMOEBA}| \approx$ -62 MV/cm) which is only slightly larger, consistent with the inclusion of polarization effects in the AMOEBA force field. Specifically, this field is 1.4-times larger than the DFT-based atomistic field ($|\vec{F}_{C\equiv N,DFT}| \approx$ -45 MV/cm; see Figure S1, left panel). This is consistent with DFT-based determinations of Stark tuning rates that are known to overestimate solvatochromic Stark tuning rates by a similar factor of ~ 1.5 (SI in ref. [26] and discussion in ref. [27]), owing to an underestimation of fields by a factor of 1.5 in this case. As such, this deviation between AMOEBA and DFT is expected. However, when using the electrostatic potential, we determined $F_{C\equiv N,DFTesp} \approx$ -150 MV/cm, an inconsistent field which is roughly 3 times larger than the other methods and Coulomb's law.

Next, we consider the determination of the electric field in the presence of an H-bonding H_2O molecule with the C≡N group of oTN (see Figure S1, right panel). According to AMOEBA, the water molecule

H-bonds with the C≡N and exerts an attractive electric field of $|\vec{F}_{C\equiv N,AMOEBA}|$ ≈ -51 MV/cm along the C≡N. In contrast, DFT determines a discrepant electric field of $|\vec{F}_{C\equiv N,DFT}|$ ≈ +10 MV/cm when using atomistic electric fields which would indicate a repulsive electrostatic interaction. We rationalize this unintuitive repulsive H-bond interaction as being due to the determination of electric fields acting on nuclei and not on the entire atom (i.e., due to the explanation provided above). Using the atomistic electrostatic potentials, we obtain $F_{C\equiv N,DFTesp}$ ≈ -44 MV/cm, an electric field which is consistent with AMOEBA.

To remain consistent with previous literature and the classical description of the VSE, we utilize the atomistic electric field definition as used in MD force fields (i.e., the formalism in eq. S4).

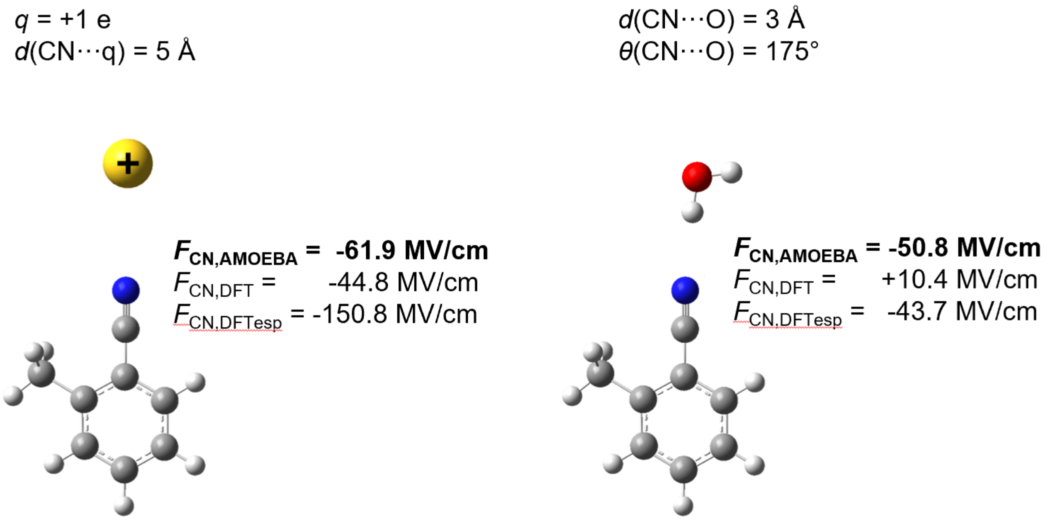

Figure S1. Comparison of AMOEBA-based and DFT-based electric fields along the C≡N bond for oTN in the presence of a charge (left) or a H-bonding water molecule (right). Left: For a classical charge (i.e., no further multipoles and van der Waals parameters used) AMOEBA and DFT provide fields of -62 and -45 MV/cm (negative sign indicates a stabilizing interaction with the C≡N). The difference by a factor of ~1.4 is consistent with previous determinations of Stark parameters using DFT (see SI text). Using the electrostatic potential to determine the electric field yields a much larger value of ~ -150 MV/cm. Right: For a H-bonding water molecule, a negative field of -51 MV/cm is found in AMOEBA (similar to the AMOEBA field with the positive charge). A similar field is obtained from the electrostatic potential (~ -44 MV/cm, smaller than the AMOEBA field by a factor of 1.2). In contrast, using atomistic electric fields from DFT yields a positive DFT-based electric field along the C≡N of ~ +10 MV/cm. The observed deviations in the DFT fields can be rationalized based on the determination of electric fields acting on the nucleus and not the entire atom (see SI text).

3. Fitting results for data in Figures 2, 3, and 4

The results from modeling DFT-based frequencies and TDMs in Figures 2 and 4 in the main text are summarized in Table S1 – Table S4; see the respective figure captions for details. Additionally, we provide a note on the accuracy of the transition dipole polarizability from fitting:

The modeling using eq. 3a in the main text resulted in diagonal tensor elements of the TDM polarizability of $A_{zz} = -0.39\,\frac{mD}{MV/cm}$, $A_{yy} = 0.00\,\frac{mD}{MV/cm}$ and $A_{xx} = -0.25\,\frac{mD}{MV/cm}$ (Table S1; see Figure 1C in the main text for the definition of x, y, and z axes). Experimentally, we obtained a linear field sensitivity of $-1.0\,\frac{mD}{MV/cm}$, which is a scalar convolution of the TDM polarizability.[9] The experimental scalar value can be compared either to the A_{zz} element, because we only evaluated electric fields along the C≡N bond (z-axis) previously, or to the trace $\mathrm{tr}(A) = -0.64\,\frac{mD}{MV/cm}$, which considers all electric field directions with equal relevance. As these two approaches will likely provide an underestimate and overestimate, respectively, we can conclude that the experimental electric field sensitivity is larger by a factor of ~ 2 in comparison to the computational prediction. This is overall a good estimate of the field sensitivity, since previous work reported that calculated VSE parameters, like the Stark tuning rate, can deviate by up to a factor of 2 depending on the level of theory.[26,28,29]

*Table S1. Optimized parameters for eq. S1 (the quadratic form of eq. 3a in the main text) for Figures 2A and 2D in the main text. Values in the row **Electrostatic** correspond to the red data points in Figures 2A and 2D; the row **Water** are the values for black data points in Figure 2A; and the row **Methanol** are the values for blue data points in Figure 2D. Entries with "0" were set to zero since they did not improve the fit further when allowed to vary and were associated with large standard errors (> 50 %).*

Perturbation	Electrostatic	Water	Methanol
$\|\vec{m}\|_0$ / mD	37.4 ± 0.1	37.6 ± 0.1	37.6 ± 0.1
A_{xx} / mD (MV/cm)$^{-1}$	-0.269 ± 0.005	-0.248 ± 0.007	-0.258 ± 0.007
A_{yy} / mD (MV/cm)$^{-1}$	0	0	0
A_{zz} / mD (MV/cm)$^{-1}$	-0.385 ± 0.012	-0.393 ± 0.007	-0.424 ± 0.009
B_{xx} / mD (MV/cm)$^{-2}$	0	0	0
B_{yy} / mD (MV/cm)$^{-2}$	0.0008 ± 0.0005	0.0064 ± 0.0004	0.0058 ± 0.0005
B_{zz} / mD (MV/cm)$^{-2}$	-0.0021 ± 0.0006	-0.0035 ± 0.0008	-0.0046 ± 0.0003
B_{xy} / mD (MV/cm)$^{-2}$	0	0	0
B_{xz} / mD (MV/cm)$^{-2}$	-0.0032 ± 0.0008	-0.0075 ± 0.0009	-0.0081 ± 0.0005
B_{yz} / mD (MV/cm)$^{-2}$	0	0	0
R^2	0.990	0.967	0.963
RMSD / mD	0.54	1.00	1.10

Table S2. Optimized parameters for eq. S2 (eq. 1a in the main text) for the red data points in Figures 2B and 2E, i.e. the vibrational frequencies under purely electrostatic perturbation. Despite the large error, $\Delta\alpha_{zz}$ was not set to zero during the fitting (as was done in Table S1), since the contribution of $\Delta\alpha_{zz}$ becomes relevant in the overall fit of eq. 7 (Figure 4A; see Table 1 in the main text).

Perturbation	Electrostatic
$\bar{\nu}_0$ / cm^{-1}	2233.0 ± 0.1
$\|\Delta\vec{\mu}\|$ / cm^{-1} (MV/cm)$^{-1}$	0.22 ± 0.01
$\Delta\alpha_{xx}$ / cm^{-1} (MV/cm)$^{-2}$	-0.0108 ± 0.0003
$\Delta\alpha_{yy}$ / cm^{-1} (MV/cm)$^{-2}$	-0.0035 ± 0.0008
$\Delta\alpha_{zz}$ / cm^{-1} (MV/cm)$^{-2}$	-0.0008 ± 0.0009
R^2	0.954
RMSD / mD	1.0

Table S3. Full set of optimized parameters from modeling the data in Figure 3A – C in the main text using eqs. 4a, 5, and 6, respectively (equations shown in table).

**Parameters**	**Optimized values**
Figure 3A - $\Delta\bar{\nu}_{HB}(d) = A \cdot (d/3Å)^{n_1}$:	
A / cm^{-1}	26.1 ± 0.2
n_1	-4.03 ± 0.07
R^2	0.99
RMSD / cm^{-1}	1.5
Figure 3A - $\Delta\bar{\nu}_{HB}(d) = A \cdot \exp(-a(d - 3Å))$:	
A / cm^{-1}	27.2 ± 0.2
a	-1.30 ± 0.02
R^2	0.99
RMSD / cm^{-1}	1.3
Figure 3B - $\Delta\bar{\nu}_{HB}(d) = A \cdot (\exp(-b(d - d_0)) - (d/d_0)^{n_2})$:	
A / cm^{-1}	45 ± 5
b / Å^{-1}	-3.1 ± 0.2
n_2	-8.2 ± 0.5
d_0 / Å	3.09 ± 0.02
R^2	0.89
RMSD / cm^{-1}	1.9
Figure 3C - $\Delta\bar{\nu}_{HB}(\theta) = B \cdot \cos[m \cdot (\theta - 180°)]$:	
B / cm^{-1}	27.8 ± 0.4
m	0.82 ± 0.01
R^2	0.89
RMSD / cm^{-1}	2.7

Table S4. Full set of optimized parameters from modeling the data in Figure 4A in the main text using eq. 2, utilizing eq.1a to describe the VSE and eq. 7 to describe the HB blueshift (overall R^2 = 0.921 and RSMD = 1.76 cm^{-1}). Note that Table 1 in the main text only reports the $\Delta\bar{\nu}_{HB}$ parameters.

**Parameters**	**Optimized values**
VSE parameters:	
$\bar{\nu}_0$ / cm^{-1}	2232.8 ± 0.1
$\lvert\Delta\vec{\mu}\rvert$ / cm^{-1} (MV/cm)$^{-1}$	0.21 ± 0.006
$\Delta\alpha_{xx}$ / cm^{-1} (MV/cm)$^{-2}$	-0.0092 ± 0.0015
$\Delta\alpha_{yy}$ / cm^{-1} (MV/cm)$^{-2}$	-0.0059 ± 0.0004
$\Delta\alpha_{zz}$ / cm^{-1} (MV/cm)$^{-2}$	-0.0019 ± 0.0003
$\Delta\bar{\nu}_{HB}$ parameters:	
d_0 / Å	3.36 ± 0.03
$\Delta\bar{\nu}_{HB}$ / cm^{-1}	16.6 ± 0.6
b / Å^{-1}	2.85 ± 0.14
m	0.91 ± 0.01
R^2	0.921
RMSD / cm^{-1}	1.76

Alternative to main text equation 7 using the exponential form in equation 4b

Based on the equally good fitting of the head-on HB distance dependence in Figure 3A using the exponential form in main text eq. 4b compared with the power law form in eq. 4a, it is possible to describe an alternative form of the HB blueshift geometry relation (main text eq. 7) using the following equation:

$$\Delta\bar{\nu}_{HB}(d,\theta) = \Delta\bar{\nu}_{HB,0} \cdot \{f_{head-on}(d) \cdot f(\theta) + f_{side-on}(d) \cdot [1 - f(\theta)]\} \quad \textit{eq. S7}$$

$$= \Delta\bar{\nu}_{HB,0} \cdot \left\{ e^{-a(d-d_0)} \cdot \cos[m \cdot (\theta - 180°)] + \left[e^{-b(d-d_0)} - \left(\frac{d}{d_0}\right)^{-8} \right] \cdot [1 - \cos[m \cdot (\theta - 180°)]] \right\}$$

The results of using eq. S7 to recapitulate our DFT-based data set are presented in Figure S2. We observe an equally excellent modeling of the HB blueshift compared with the results from using eq. 7 (main text Figure 4), with slightly improved R^2 and RMSD values in H-bonding environments due to the fact that an additional open parameter is used during the fitting (d_0 is the only open parameter in the power law form; a and d_0 are open in the exponential form). However, we note that the exponential form in eq. 4b would imply blueshifts for head-on nitrile H-bonds are dominated by Pauli repulsion, but we show evidence in SI Section 4 that this is unlikely to be the origin of the blueshift for these configurations.

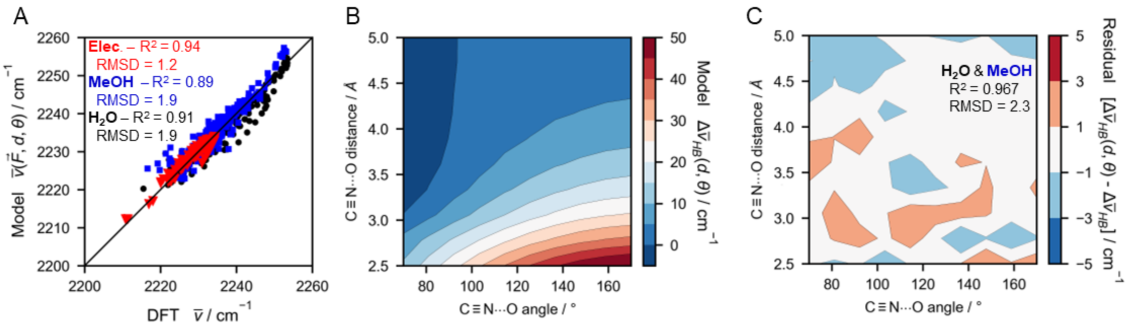

Figure S2. Alternative modeling to Figure 4 in the main text using eq. S7 with the exponential form of the head-on HB distance dependence. A: Correlation plot of modeled $\bar{\nu}(\vec{F}, d, \theta)$ (eq. 2 utilizing eq. 1a and eq. s7) and DFT-based vibrational frequencies shows that the model applies equally well for purely electrostatic perturbations (red triangles) and in the presence of water and methanol HBs (black circles and blue squares, respectively) with an overall R^2 = 0.92 and RMSD = 1.8 cm^{-1} (R^2 and RMSD values for each distinct environment are shown in the graph). Fitting parameters for the model are reported in Table S5. B: 2D heat plot of $\Delta\bar{\nu}_{HB}(d,\theta)$ with water and methanol as HB donors according to the model in eq. s7. C: 2D heat plot of the residuals between modeled $\Delta\bar{\nu}_{HB}(d,\theta)$ (see B) and $\Delta\bar{\nu}_{HB}$ from main text Figure 2C and F (R^2 = 0.967 and RMSD = 2.3 cm^{-1}).

Table S5. Full set of optimized parameters from modeling the data in Figure 4A in the main text using eq. 2, utilizing eq. 1a to describe the VSE and the alternative to eq. 7, i.e. eq. S7, to describe the HB blueshift (overall R^2 = 0.921 and RSMD = 1.76 cm^{-1}).

**Parameters**	**Optimized values**
VSE parameters:	
$\bar{\nu}_0$ / cm^{-1}	2232.8 ± 0.1
$\lvert\Delta\vec{\mu}\rvert$ / cm^{-1} $(MV/cm)^{-1}$	0.21 ± 0.006
$\Delta\alpha_{xx}$ / cm^{-1} $(MV/cm)^{-2}$	-0.0095 ± 0.0015
$\Delta\alpha_{yy}$ / cm^{-1} $(MV/cm)^{-2}$	-0.0061 ± 0.0004
$\Delta\alpha_{zz}$ / cm^{-1} $(MV/cm)^{-2}$	-0.0019 ± 0.0003
$\Delta\bar{\nu}_{HB}$ parameters:	
d_0 / Å	3.32 ± 0.04
$\Delta\bar{\nu}_{HB}$ / cm^{-1}	18.5 ± 0.6
a / $Å^{-1}$	1.29 ± 0.09
b / $Å^{-1}$	2.88 ± 0.14
m	0.90 ± 0.01
R^2	0.927
RMSD / cm^{-1}	1.72

4. Testing multipolar or Pauli repulsion in head-on or side-on HBs

Higher order multipole effects can be destabilizing for head-on nitrile – water H-bond interactions

In support of our suggestion that the HB blueshift in head-on HB geometries could originate from higher multipole interactions like dipole-quadrupole interactions, we performed an energy decomposition analysis for oTN-H_2O complexes with C≡N-H_2O head-on (3.0 Å at 175°) and side-on HBs (3.0 Å at 80°; and 3.5 Å at 70°). For this analysis, Tinker's ANALYZE function was used together with key-files, where all oTN parameters were zeroed out except for the parameter of interest (Table S6.). While we report the atomistic contributions of C and N atoms of the nitrile for the interested reader, we only discuss the summed contributions as these are most pertinent for considering the C≡N oscillator used within the VSE framework. We find that polarization of the C≡N (polarizability parameters on C and N) has a (stabilizing) negative energetic contribution in line with the attractive nature of head-on and side-on C≡N-H_2O HBs. The same is true for the summed contributions of all multipole parameters (charge, dipole and quadrupole) on the C and N atoms; this contribution is 3 – 5 times larger than the one due to polarizability. When decomposing the multipole interaction energy into the individual contributions, we note that the partial charges of C and N are the major contributors to the attractive C≡N-H_2O HBs. Since the charge separation between C and N is what underlies the C≡N dipole to the largest extent, these interactions are the origin of the VSE of the C≡N group, and the (stabilizing) negative energetic contribution is consistent with the redshift described by eq. 1 in the main text. In contrast, we find that the dipole and quadrupole parameters of C and N can contribute a positive energetic contribution, i.e. a repulsive interaction. As the charge difference between C and N leads to a dipole, a separation between the C's and N's dipole leads to (an averaged dipole and) a quadrupolar behavior; analogously, the atomistic quadrupoles will contribute to (an averaged quadrupole and) higher multipolar behavior. As such, we observe that higher order multipole interactions than the "electric field-dipole" interaction of the C≡N-H_2O HB can indeed lead to a repulsive interaction. This repulsive interaction may be the origin of the d^{-4} dependence of the head-on HB blueshift (see main text eq. 4a). However, as mentioned in the main text, convolution with some Pauli repulsion contributions cannot be excluded.

Table S6. Energy decomposition of the H-bonding C≡N-H_2O interaction for head-on (3 Å / 175°) and side-on configurations (3.0 Å / 70°; 3.5 Å / 80°), determined using the ANALYZE function in Tinker. See SI text for a description of how these energies were determined.

**Geometry**	**3.0 Å / 175° (values in kcal/mol)**	**3.0 Å / 70° (values in kcal/mol)**	**3.5 Å / 80° (values in kcal/mol)**
**C≡N group:**			
C≡N polarization	-1.3483	-0.8908	-0.8234
C≡N multipoles	-6.1771	-2.6885	-2.1862
C≡N charge	-7.2872	-4.3185	-3.261
C≡N dipole	4.9376	1.072	0.3078
C≡N quadrupole	-3.8275	0.5581	0.767
**Per atom:**			
N charge	-11.2648	-4.5596	-5.1812
N dipole	5.0405	1.2144	0.4339
N quadrupole	-3.4476	0.7505	0.6928
C charge	3.9776	0.2411	1.9202
C dipole	-0.1029	-0.1424	-0.1261
C quadrupole	-0.3799	-0.1924	0.0742

Testing the contribution of Pauli repulsion in the H-bond blueshift

As discussed in the main text, the exponential fit of the head-on distance dependence of the HB blueshift in Figure 3A yields an exponential decay constant of a ≈ 1.3 Å^{-1} (see Table S5), which is inconsistent with values from MD force fields of molecular solids.[30] In fact, the value of 1.3 Å^{-1} would indicate a much longer ranging Pauli repulsion effect than what is expected. To clarify if Pauli repulsion can contribute to the blueshift, we performed DFT simulations (similar level of theory as described in SI Section 1) where we approached the C≡N group with a Ne atom from head-on (175°) or side-on directions (80°) from a distance of 5 to 2.5 Å (the same range as we used in the main text). Due to being closed shell and charge neutral, the Ne atom serves as a perturbation which should only interact via van der Waals interactions and Pauli repulsion. For the head-on approach (Figure S3 left panel), we observe a slight *redshift* of the DFT-derived wavenumber of the C≡N stretch by ~ -2 cm^{-1} below N---Ne distances of 3.25 Å. For comparison, we used the same geometries to determine the electric field on the C≡N from AMOEBA. Using these electric fields and our VSE parameters from Table S4, we also find a very small redshift (~ -0.5 cm^{-1}) due to the mutual polarization between oTN and Ne. Importantly, in main text Figure 3A we observe a HB blueshift of +50 cm^{-1}, which is not reproduced by the Ne atom approach. This suggests that the HB blueshift in head-on configurations is not due solely to Pauli repulsion.

Furthermore, we repeated this approach for a side-on configuration, where we see a blueshift by +6 cm^{-1} at a Ne – C≡N distance of 2.5 Å, even though the VSE would predict a very small redshift (< 0.5 cm^{-1}) at this distance. This result supports Pauli repulsion being responsible for the HB blueshifts in side-on geometries.

As a point of clarification, the DFT frequency shifts observed due to the Ne atom should not be compared on an absolute scale with the HB blueshifts in the main text due to different van der Waals radii and polarizabilities of the H-bonding OH groups in water and methanol. Nevertheless, this exercise serves as a simple demonstration for when Pauli repulsion becomes relevant.

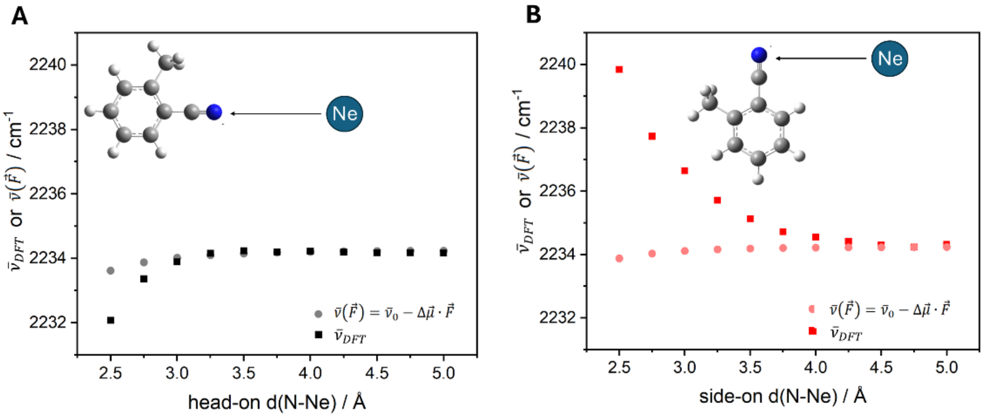

Figure S3. DFT-based test of potential Pauli repulsion contributions for head-on (A) and side-on (B) interaction geometries of the C≡N group with a Ne atom. The closed shell Ne atom serves to induce a van der Waals and Pauli repulsion perturbation to the C≡N with minimal electrostatic interaction. A: For a head-on approach, we note that vibrational frequencies from DFT show small redshifts by ~ 2 cm^{-1} at short distances. The VSE shifts (calculated using AMOEBA electric fields and parameters in Table S4) also show a small redshift due to mutual polarization. The redshifts suggest that there are no considerable repulsive interactions of the C≡N with the Ne in the head-on configuration. B: For the side-on approach, we note a considerable blueshift from DFT suggesting repulsive forces such as Pauli repulsion are relevant. As before, the VSE predicts a small redshift due to polarization, but that is not observed this time.

5. Alternative models to fit the distance dependence for side-on HBs

In order to fit the asymptotic drop of $\Delta\bar{\nu}_{HB,0}$ with the well at ~ 3.5 Å for side-on HBs (Figure 3B in the main text), we tested various models, including:

- a generalized Lennard-Jones-like function (Figure S4A):

$$\Delta\bar{\nu}_{HB,side-on}(d) = \Delta\bar{\nu}_{HB,0}\left[\left(\frac{d}{d_0}\right)^{n_1} - \left(\frac{d}{d_0}\right)^{n_2}\right] \quad \textit{eq. S8}$$

- a Morse function (Figure S4B):

$$\Delta\bar{\nu}_{HB,side-on}(d) = \Delta\bar{\nu}_{HB,0}[1-\exp(-b(d-d_0))]^2 \quad \textit{eq. S9}$$

- a buffered-7-14 function (Figure S4C):

$$\Delta\bar{\nu}_{HB,side-on}(d) = \Delta\bar{\nu}_{HB,0}\left[\frac{1+b_1}{\frac{d}{d_0}+b_1}\right]^7\left[\frac{1+b_2}{\left(\frac{d}{d_0}\right)^7+b_2}-2\right] \quad \textit{eq. S10}$$

- a Buckingham-like function (Figure S4D, as shown in the main text, Figure 3B and eq. 5)

$$\Delta\bar{\nu}_{HB,side-on}(d) = \Delta\bar{\nu}_{HB,0}\left[e^{-b(d-d_0)} - \left(\frac{d}{d_0}\right)^n\right] \quad \textit{eq. S11}$$

Figure S4 shows the results of these four models. Each of the models fit the data with $R^2 > 0.83$, with the Buckingham-like function giving the best result with $R^2 = 0.89$ due to a better description of the well at 3.5 Å and subsequent rise in blueshift values at larger HB distances.

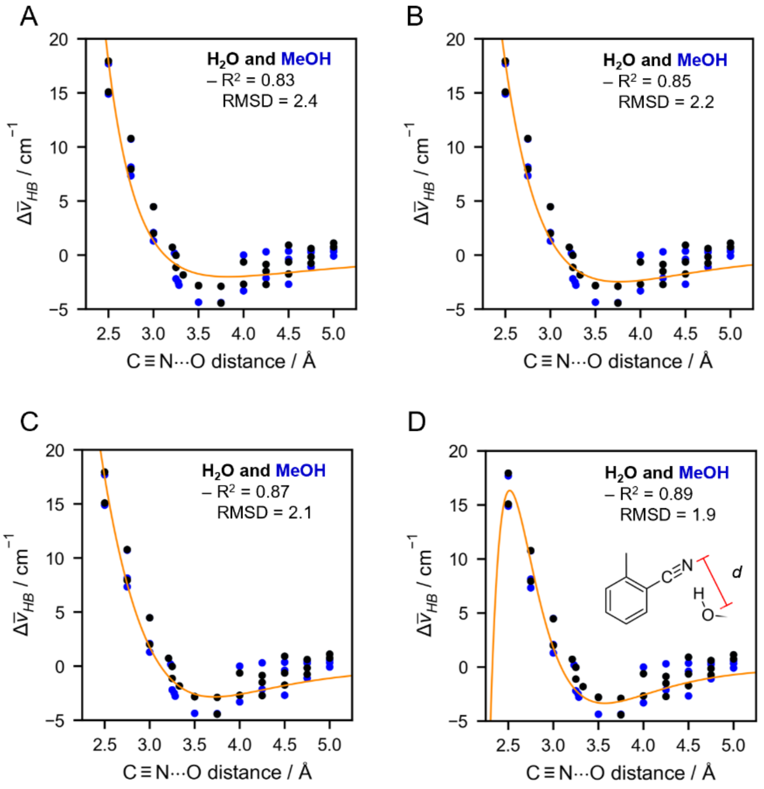

Figure S4. Tests of the functional forms in eq. S8 - eq. S11 to model $\Delta\bar{\nu}_{HB}$ for side-on HBs (panel D is the same fit as shown in Figure 3B). From A (Lennard-Jones equation), to B (Morse equation), to C (buffered 7-14 equation), to D (Buckingham equation), the fit improves from R^2 of 0.83 to 0.89, with modeling of the well at 3.5 Å most accurately described in D.

6. Parameter m in angular dependence of the HB blueshift

The angular dependence of the HB blueshift was modeled using eq. 5 in the main text:

$$f(\theta) = \cos[m \cdot (\theta - 180°)] \qquad \textit{eq. S12}$$

where θ = 180° is a head-on HB and m is a parameter that modulates when $f(\theta)$ = 0. The inclusion of m can be rationalized based on the fact that we express HB distances as the $N_{C≡N}$ – $O_{HB\ donor}$ distance and HB angles as the $C_{C≡N}$ – $N_{C≡N}$ – $O_{HB\ donor}$ angle (d and θ in Figure S5A). We chose this representation because it is consistent with how HBs are described in radial distribution functions (i.e. using heavy atom distances). However, the dipolar VSE – and higher multipolar interactions – utilize the C≡N-group (i.e. its center) as the interaction site and not the $N_{C≡N}$ atom (Figure S5B). In this representation, the distance and angle change to d' and θ'. The relation between the two angles is given by:

$$\tan(180° - \theta') = \frac{d \cdot \sin(180° - \theta)}{d \cdot \cos(180° - \theta) + \frac{b_{C≡N}}{2}} \qquad \textit{eq. S13}$$

according to Figure S5C, where $b_{C≡N}$ is the C≡N bond length. With a bond length of $b_{C≡N}$ = 1.156 Å (determined from our DFT calculations with oTN in vacuo) we determined that a side-on HB, i.e. with θ' = 90°, occurs at an angle of $\theta \approx$ 80° when $N_{C≡N}$ – $O_{HB\ donor}$ distances of d = 3.0 Å – 3.5 Å are adopted. The ratio between these two angles is the parameter $m = \frac{\theta}{\theta'} = \frac{80°}{90°} \approx 0.9$, which is close to the fitted value in the main text.

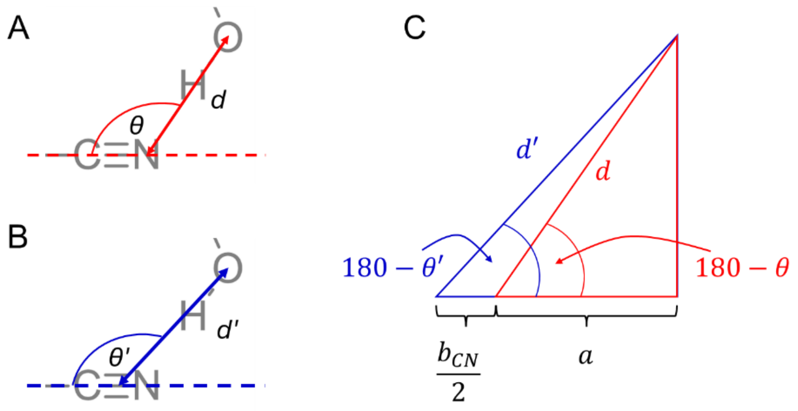

Figure S5. Difference in HB angles with respect to the C≡N bond axis, when taking (A) the $N_{C≡N}$ atom or (B) the C≡N bond center as the interaction site. In the main text, we use the $N_{C≡N}$ atom as the interaction site such that HB distance and angle are defined as shown in A. (C) indicates the "translation" between the two representations.

7. Modeling HB blueshifts of two simultaneous HBs

Table S7. Results from DFT calculations of oTN with two simultaneous HBs to two individual water molecules (indices "Water,1" and "Water,2") with restrained θ(C≡N---$O_{water,i}$) (with i = 1,2) angles and distances allowed to optimize. $\Delta\bar{\nu}_{HB,DFT}$ was calculated as described in the main text as the difference between DFT-based vibrational frequencies from normal mode analysis and the electric field dependence (i.e. dipolar VSE effect only) of the vibrational frequency (shown in main text Figure 2B, E). $\Delta\bar{\nu}_{HB,model}$ was obtained as $\Delta\bar{\nu}_{HB,model} = \Delta\bar{\nu}_{HB,model,1}(d_{Water,1}, \theta_{Water,1}) + \Delta\bar{\nu}_{HB,model,2}(d_{Water,2}, \theta_{Water,2})$, where each contribution is calculated using eq. 7 in the main text with corresponding parameters from Table S4.

$\theta_{Water,1}$ / °	$d_{Water,1}$ / Å	$\theta_{Water,2}$ / °	$d_{Water,2}$ / Å	$\Delta\bar{\nu}_{HB,DFT}$ / cm^{-1}	$\Delta\bar{\nu}_{HB,model}$ / cm^{-1}
80	3.15	70	3.38	-12.46	-1.19
90	3.07	70	3.39	-8.27	3.33
80	3.15	80	3.17	-0.63	3.19
100	3.01	70	3.37	-0.38	7.91
90	3.07	80	3.17	4.77	7.72
110	2.97	70	3.35	7.97	12.11
100	3.01	80	3.17	12.40	12.26
90	3.07	90	3.05	12.79	12.86
120	2.97	70	3.34	14.62	15.27
100	3.03	100	3.03	20.91	20.54
110	2.98	80	3.15	21.09	16.39
170	2.98	70	3.35	23.13	23.98
160	2.95	70	3.33	24.20	24.17
140	2.94	130	3.49	27.34	33.85
120	3.30	120	2.92	27.89	29.94
110	2.98	100	3.04	28.47	24.58
130	3.36	130	2.93	29.52	33.51
110	2.98	110	3.03	30.49	28.05
130	2.92	120	3.32	31.00	32.34
120	2.99	110	3.03	33.28	30.73
150	2.92	120	4.83	33.37	28.58
150	2.92	130	4.85	36.95	29.31
170	2.94	120	3.41	37.10	36.86
170	2.96	80	3.17	37.76	28.79
160	2.90	80	3.15	38.30	30.43
160	2.92	130	4.82	40.87	30.83
170	2.92	90	3.04	42.99	35.76

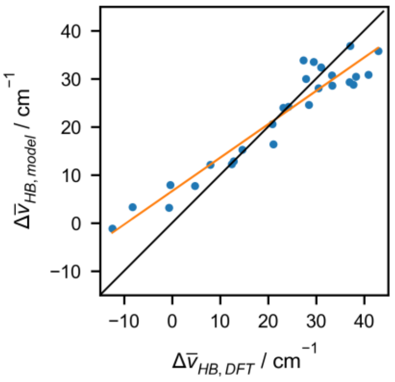

Figure S6. Correlation between modeled (eq. 7) and DFT-based $\Delta\bar{\nu}_{HB}$ values for two simultaneous nitrile HBs with two individual water molecules (see Table S7 for data). The Orange line is a linear fit to the data with $\Delta\bar{\nu}_{HB,model} = (0.69 \pm 0.04) \cdot \Delta\bar{\nu}_{HB,DFT} + (6.6 \pm 1.2)$ and R^2 = 0.982. The black line represents a perfect correlation, i.e. with slope of 1 and through the origin, coinciding well with data points > 10 cm^{-1}; data points < 10 cm^{-1} are overestimated by the additive model.

8. Angle dependence for common HB distances in solvents – radial distribution functions of ortho-tolunitrile

We narrowed down Figure 4B in the main text to a relevant regime of commonly adopted HB geometries in solvents for heavy atom distances of < 4.0 Å. Based on AMOEBA MD simulations of oTN in water and methanol, the average HB distance decreases monotonically from 3.35 Å for side-on HBs (70°) to 2.93 Å when head-on HBs are adopted (Figure S7A). These typical HB distances are indicated in the 2D heat plot of the geometry-dependence of the HB blueshift in Figure S7B. Extracting the HB blueshift values along the line of average HB distances (and the width of the distribution in Figure S7A), we note $\Delta\bar{\nu}_{HB} \approx$ -5 cm^{-1} for side-on HBs interacting with the C≡N's π-orbitals [~70° for θ(C≡N---O)] (Figure S7C). As the angle and distance concomitantly increase and decrease, respectively, the blueshift increases steadily, plateauing around 26 cm^{-1} for head-on HBs with θ(C≡N---O) > 170°. The data points of our test to experimental data are included in Figure S7C. F92oCNF shows an excellent match of experimental $\Delta\bar{\nu}_{HB}$ to predicted values; MOFs are all above the generalized trend, which is expected since the HB distances are shorter than the predicted value from the trend line given the nitrile HB angles (see specific values in the main text). F28oCNF, oTN in water, and oTN in MeOH are far below the line due to the presence of fluctuating HBs as discussed in the main text.

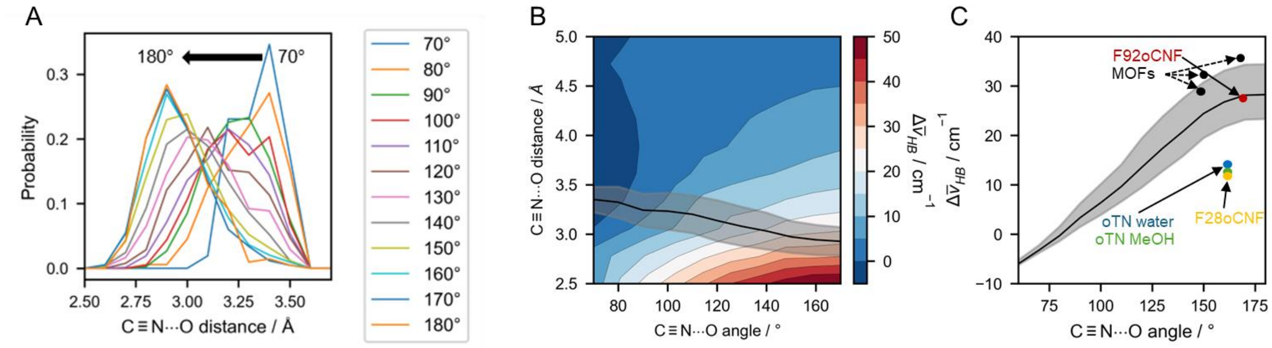

Figure S7. A: C≡N---$O_{water/methanol}$ angle-dependent HB heavy atom distances of oTN in water and methanol obtained from AMOEBA MD simulations (see Methods Section). The distance distributions were determined at angles specified in the legend (bins were defined as bin centers ±5°). B: 2D heat plot of $\Delta\bar{\nu}_{HB}$ (water and methanol as HB donors) determined from DFT-based nitrile frequencies minus pure VSE shifts (parameters in Table S4; see Figure 4 in the main text). The black line indicates the average HB distance taken from A. The gray shaded area corresponds to the width of the distributions in A (1σ). C: Angle dependence of $\Delta\bar{\nu}_{HB}$ at corresponding average HB distance (see A and B); gray shaded area corresponds with the gray shaded area in B. The F92oCNF data point falls on the trend line, but the points for F28oCNF, oTN in water, and oTN in methanol do not due to fast fluctuations in their H-bonding interactions with the nitrile (see SI Section 10). MOFs appear above the line because of the presence of short HBs (~ 2.8 Å) but are still consistent with our modeling when accounting for this shorter HB distance (see very good correlation between predicted and experimental $\Delta\bar{\nu}_{HB}$ in Figure 7 in the main text).

9. HB analysis of MD trajectories: oTN in water and MeOH and oCNF in PYP

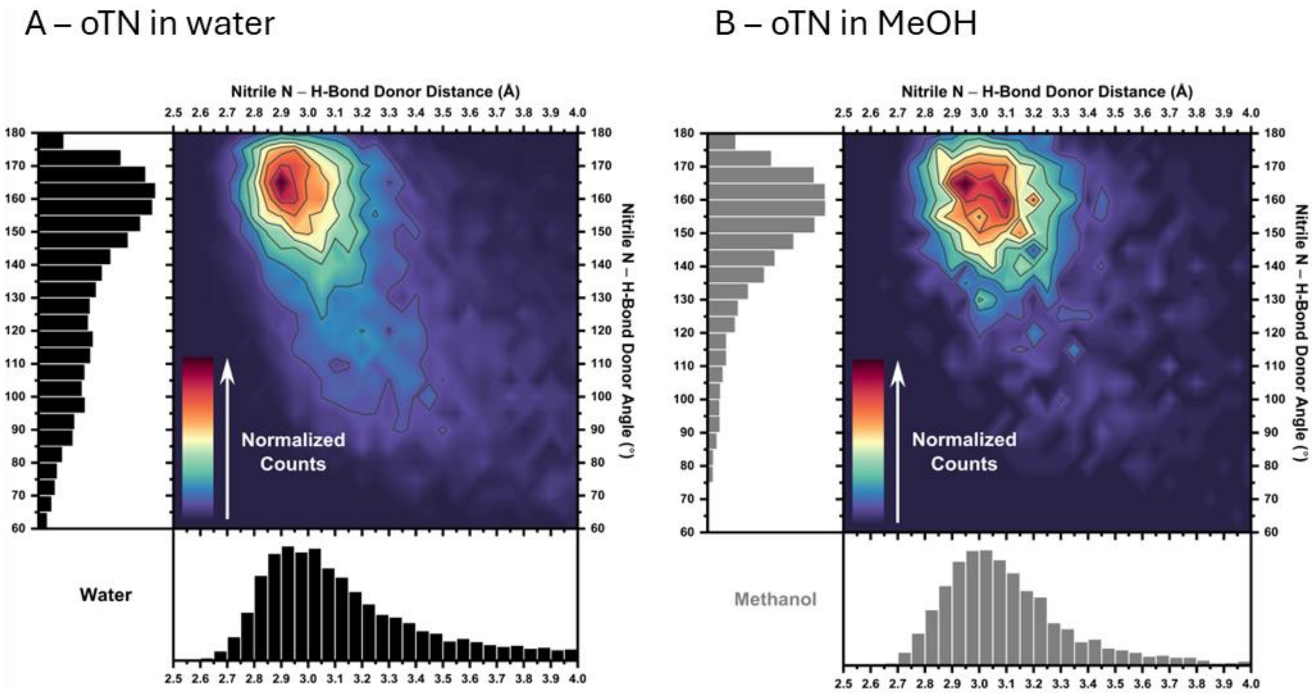

Figure S8. Nitrile H-bond angle vs distance for oTN in water (A) or oTN in methanol (B) depicted as a contour plot describing H-bond angle/distance sampling space. 1D histograms show the sampling space projected along the individual variables. See Table S8 for average values and width of the distributions.

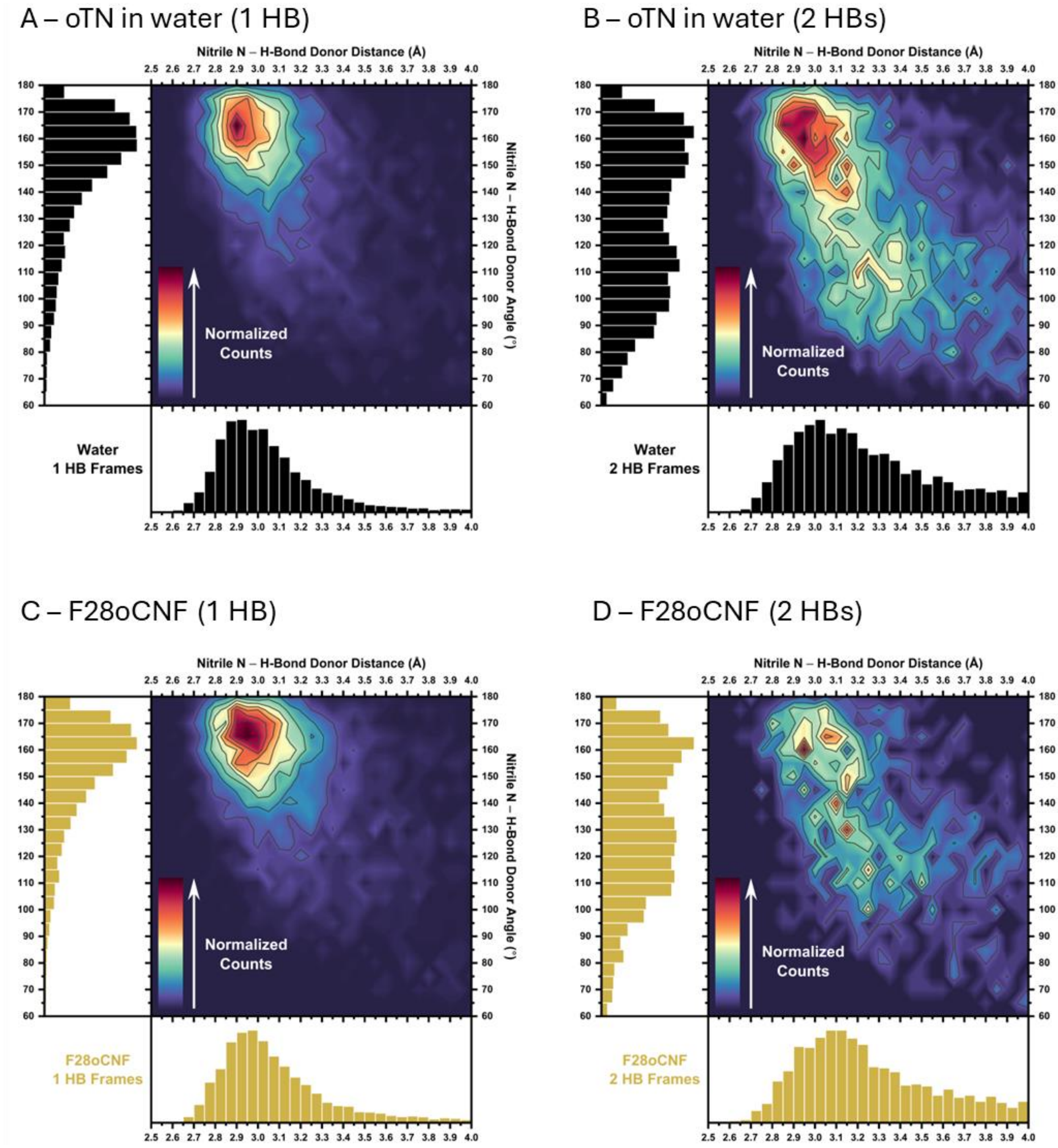

Figure S9. Nitrile H-bond angle vs distance for oTN in water (A, B) or F28oCNF PYP (C, D) depicted as a contour plot describing H-bond angle/distance sampling space for the fractions of single H-bonded (A, C) and double H-bonded nitriles (B, D). 1D histograms show the sampling space projected along the individual variables. See Table S8 for average values and widths of the distributions.

To extract average H-bond donor distances and angles from AMOEBA MD simulations for the contour plots in Figure S8 and Figure S9, the plots were fit with a rotated Gaussian surface (*i.e.*, a 2D Gaussian):

$$C = A \exp\left\{-\frac{1}{2}\left(\frac{d_{NX}\cos(\varphi)+\theta_{CNX}\sin(\varphi)-\mu_{d_{NX}}\cos(\varphi)+\mu_{\theta_{CNX}}\sin(\varphi)}{\sigma_{d_{NX}}}\right)^2-\frac{1}{2}\left(\frac{-d_{NX}\sin(\varphi)+\theta_{CNX}\cos(\varphi)-\mu_{d_{NX}}\sin(\varphi)+\mu_{\theta_{CNX}}\cos(\varphi)}{\sigma_{\theta_{CNX}}}\right)^2\right\}$$

eq. S14

where *C* is the relative number of counts, *A* is a scaling factor, d_{NX} is the H-bond donor distance, θ_{CNX} is the H-bond donor angle, φ is the rotation angle in the plane of the contour plot, $\mu_{d_{NX}}$ is the H-bond distance mean, $\mu_{\theta_{CNX}}$ is the H-bond angle mean, $\sigma_{d_{NX}}$ is the H-bond donor standard deviation, and $\sigma_{\theta_{CNX}}$ is the H-bond angle standard deviation. Similar to our previous work,[14] the distributions for oTN in water and MeOH (Figure S8A, B) were each modeled with a single 2D Gaussian; the fitting results are shown in Table S8, along with the results from our previous work for the PYP variants F28oCNF and F92oCNF.

Since the plots for oTN in water (Figure S8A) and F28oCNF (Figure S20 in ref. [14]) demonstrate two populations with respect to the HB angles, we performed further analysis to better understand the origin of these populations (with resulting fits provided in Table S8). We classified the AMOEBA MD trajectory into H-bonding and non-H-bonding populations by defining a HB threshold of 4.0 Å HB distance and using a 30° HB cone (using a 3.5 Å HB distance provided similar results as discussed in ref. [14]). H-bonding frames were further separated into those with one or two nitrile HBs, and contour plots for the one and two HB populations are shown in Figure S9. Comparison of the one and two HB plots indicates that for two HBs, two populations are observed with respect to the HB angles, while for one nitrile HB, a single population is observed; consequently, we performed single 2D Gaussian fits for the one HB populations and fits with two 2D Gaussians for the two HB populations. Comparison of the one and two HB contour plots makes clear that the HB angle heterogeneity in Figure S8A (and Figure S20 in ref. [14]) arises from sampling in two nitrile HB configurations, where the second HB donor adopts longer distances and shallower angles while the first HB donor adopts a more ideal H-bonding geometry.

Table S8. 1D histograms in Figure S8 and Figure S9 were modeled using a single Gaussian for the entire distributions (Figure S8) or as one or two Gaussians for distributions which were separated into single and double H-bonded fractions, respectively (Figure S9). Average H-bond distances and angles are the peak positions of the Gaussian(s); standard deviations (s.d.) are the width(s) of the Gaussians. Plus and minus values are the standard errors of the fits, with the number of H-bonding frames > 2000 for all trajectories.

oTN Environment	Avg. H-Bond Distance, $\mu_{d_{NX}}$ (Å)	Avg. H-Bond Angle, $\mu_{\theta_{CNX}}$ (°)	H-Bond Distance s.d., $\sigma_{d_{NX}}$ (Å)	H-Bond Angle s.d., $\sigma_{\theta_{CNX}}$ (°)	R^2
Fits with a single Gaussian:					
Water	2.98 ± 0.003	160.4 ± 0.7	0.16 ± 0.003	24.2 ± 0.7	0.82
Methanol	3.04 ± 0.003	159.7 ± 0.3	0.16 ± 0.003	15.5 ± 0.3	0.89
F28oCNF[a]	3.00 ± 0.003	162.9 ± 0.3	0.15 ± 0.003	16.4 ± 0.4	0.88
F92oCNF[a]	2.93 ± 0.001	167.2 ± 0.1	0.13 ± 0.001	9.9 ± 0.1	0.96
[a] taken from ref. [14]					
Fits to one and two HB populations:					
oTN in Water with 1HB					
Population 1	2.97 ± 0.002	161.8 ± 0.3	0.15 ± 0.002	15.3 ± 0.3	0.89
oTN in Water with 2HBs					
Population 1	2.99 ± 0.01	161.9 ± 0.5	0.14 ± 0.004	13.6 ± 0.5	0.85
Population 2	3.30 ± 0.01	116.2 ± 1.1	0.30 ± 0.008	25.6 ± 1.0	
F28oCNF with 1HB					
Population 1	2.99 ± 0.003	163.5 ± 0.2	0.15 ± 0.002	14.7 ± 0.3	0.91
F28oCNF with 2HBs					
Population 1	3.03 ± 0.01	158.4 ± 1.3	0.16 ± 0.007	20.4 ± 1.5	0.66
Population 2	3.41 ± 0.03	116.1 ± 1.4	0.32 ± 0.02	17.9 ± 1.1	

10. HB analysis of MD trajectories: HB residence times

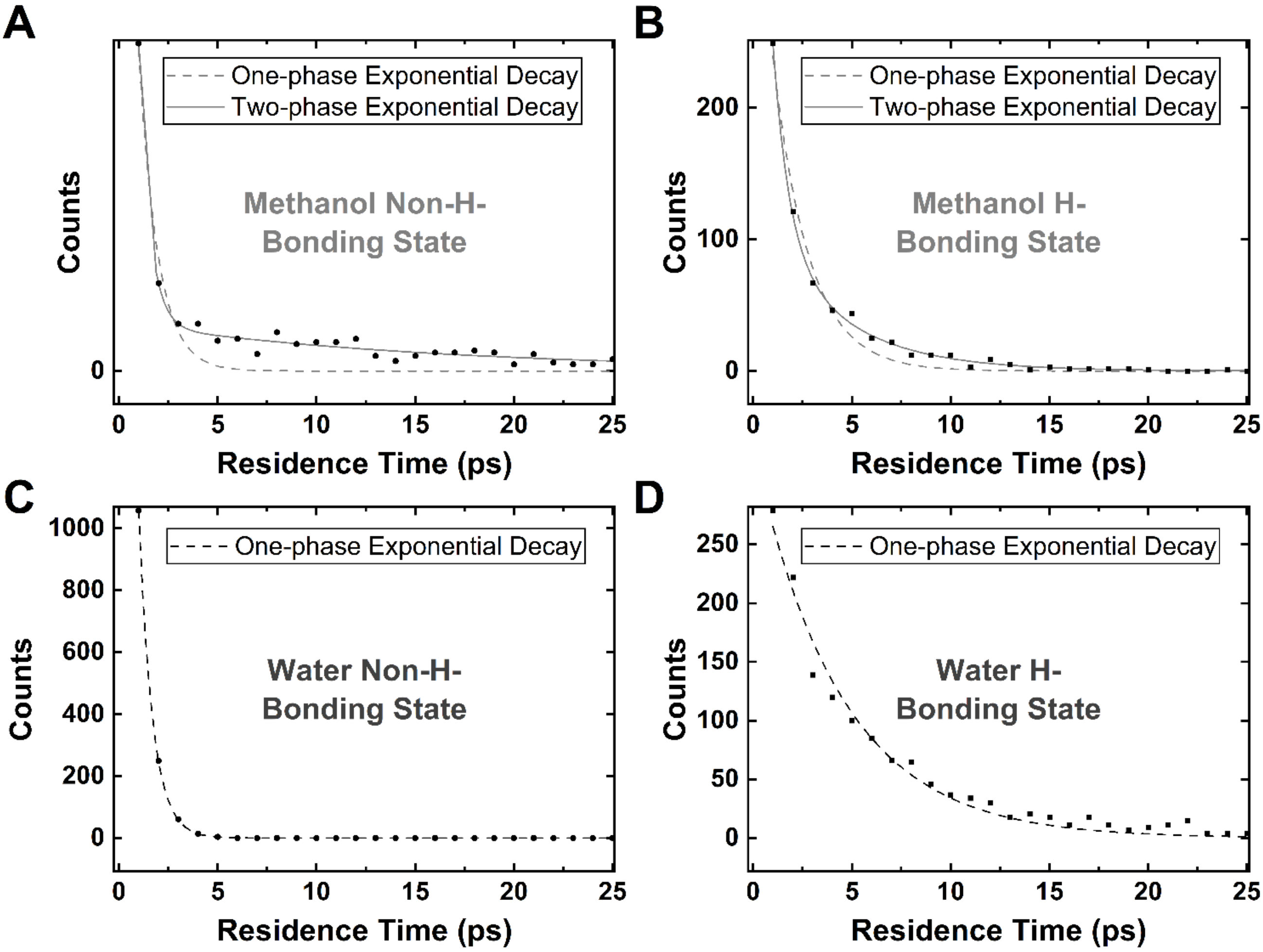

Figure S10. oTN H-bonding/non-H-bonding residence times for methanol (A/B) and water (C/D) with states output every 1 ps; the residence times were extracted using statistics from the entire MD simulations. Fits with a single exponential are shown as dashed lines; biexponential fits are shown as solid lines. For oTN in methanol (A and B), both the non-H-bonding and H-bonding residence times required biexponential fits to the distributions (i.e. single exponentials were poor models of the data), indicative of both fast and slow H-bonding ←→ non-H-bonding exchange (the slow exchange will lead to two distinct bands in IR spectra; see SI Section 11). In contrast, residence times for the H-bonding and non-H-bonding states for oTN in water (C, D) are well fit using single exponentials. Similar plots for F92oCNF and F28oCNF are found in ref. [14]. Results from the fits are shown in Table S9.

Single or double decaying exponentials fits to the plots in Figure S10 have the form:

$$c = A_1 exp\left(\frac{-t_r}{\tau_1}\right) + A_2 exp\left(\frac{-t_r}{\tau_2}\right) \qquad \textit{eq. S15}$$

where c is the number of counts, t_r is the residence time, and A_1/A_2 and τ_1/τ_2 are the amplitudes and lifetimes, respectively, of the first and second exponentials.

Table S9. Results from exponential fits for non-H-bonding and H-bonding nitrile populations for oTN in methanol (MeOH), oTN in water (see Figure S10 for both), F28oCNF, and F96oCNF (see ref. [14] for both). oTN H-bonding/non-H-bonding states were output every 1 ps, while PYP states were output every 10 ps[14] (this discrepancy comes from the longer simulation times needed to sample the protein degrees of freedom, which requires decreased state sampling for a given output file size). Monoexponential or biexponential fits are indicated in the table. Values for A_i (see eq. S15) are scaled to a total sum of 1000 transitions for comparison among the fits. Note that low numbers for A_i correlate with long τ_i values, i.e. long residence time is associated with infrequent transitions; low values for A_i should not be interpreted as transitions of low relevance or that are negligible – see Figure S8 and Figure S9 for a visual representation of this argument.

System	State	A_1	τ_1 (ps)	A_2	τ_2 (ps)	R^2
Monoexp.:						
MeOH	Non-H-Bonding	560 ± 58	0.96 ± 0.07			0.90
MeOH	H-Bonding	440 ± 98	1.80 ± 0.05			0.98
Biexp.:						
MeOH	Non-H-Bonding	603 ± 20	0.55 ± 0.03	16.8 ± 0.3	15.8 ± 1.0	0.99
MeOH	H-Bonding	307 ± 0.4	0.81 ± 0.04	72.8 ± 0.2	3.9 ± 0.2	0.998
Monoexp.:						
Water	Non-H-Bonding	931 ± 1	0.695 ± 0.001			1
Water	H-Bonding	69 ± 4	4.4 ± 0.1			0.99
Monoexp.:[a]						
F28oCNF	Non-H-Bonding	690 ± 7	11.1 ± 0.1			1
F28oCNF	H-Bonding	310 ± 3	19.8 ± 0.2			0.998
Biexp.:[a]						
F92oCNF	Non-H-Bonding	518 ± 96	3.8 ± 0.3	9.2 ± 0.6	81 ± 5	0.99
F92oCNF	H-Bonding	472 ± 32	4.6 ± 0.3	1.6 ± 0.2	343 ± 46	1

[a] taken from ref. [14]

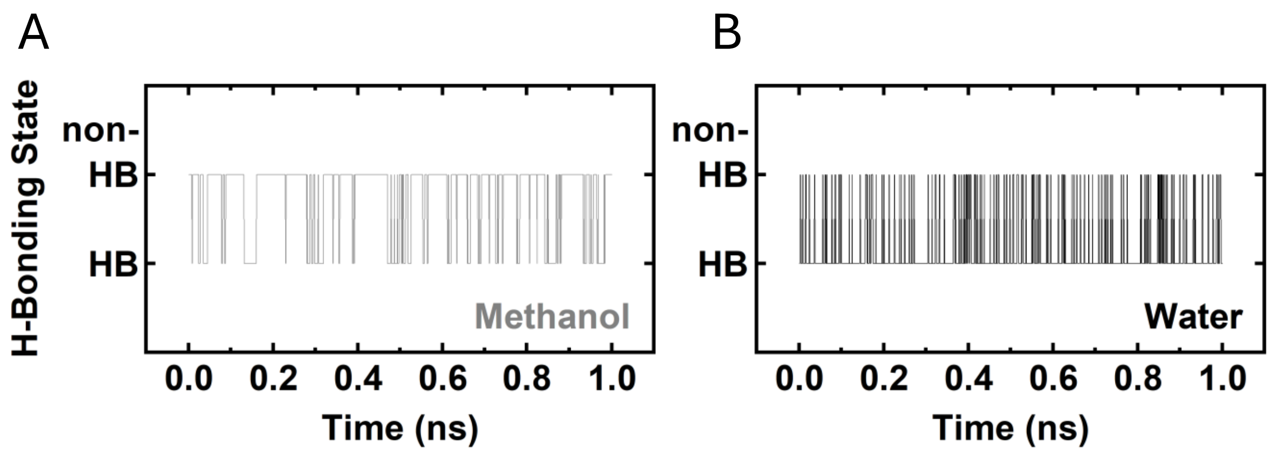

Figure S11. oTN H-bonding/non-H-bonding states as functions of time with outputs every 1 ps using the first 1 ns of the small molecule runs. The traces provide a qualitative assessment of Figure S10's results. A: The C≡N of oTN in methanol typically resides for a long time in the non-H-bonding state; in the H-bonding state, residence times are typically shorter, but occasional "long" residence times occur (see the time between 0.1 and 0.2 ns). B: In contrast, water exists in the non-H-bonding state very transiently and extended residence times are only seen for the H-bonding state.

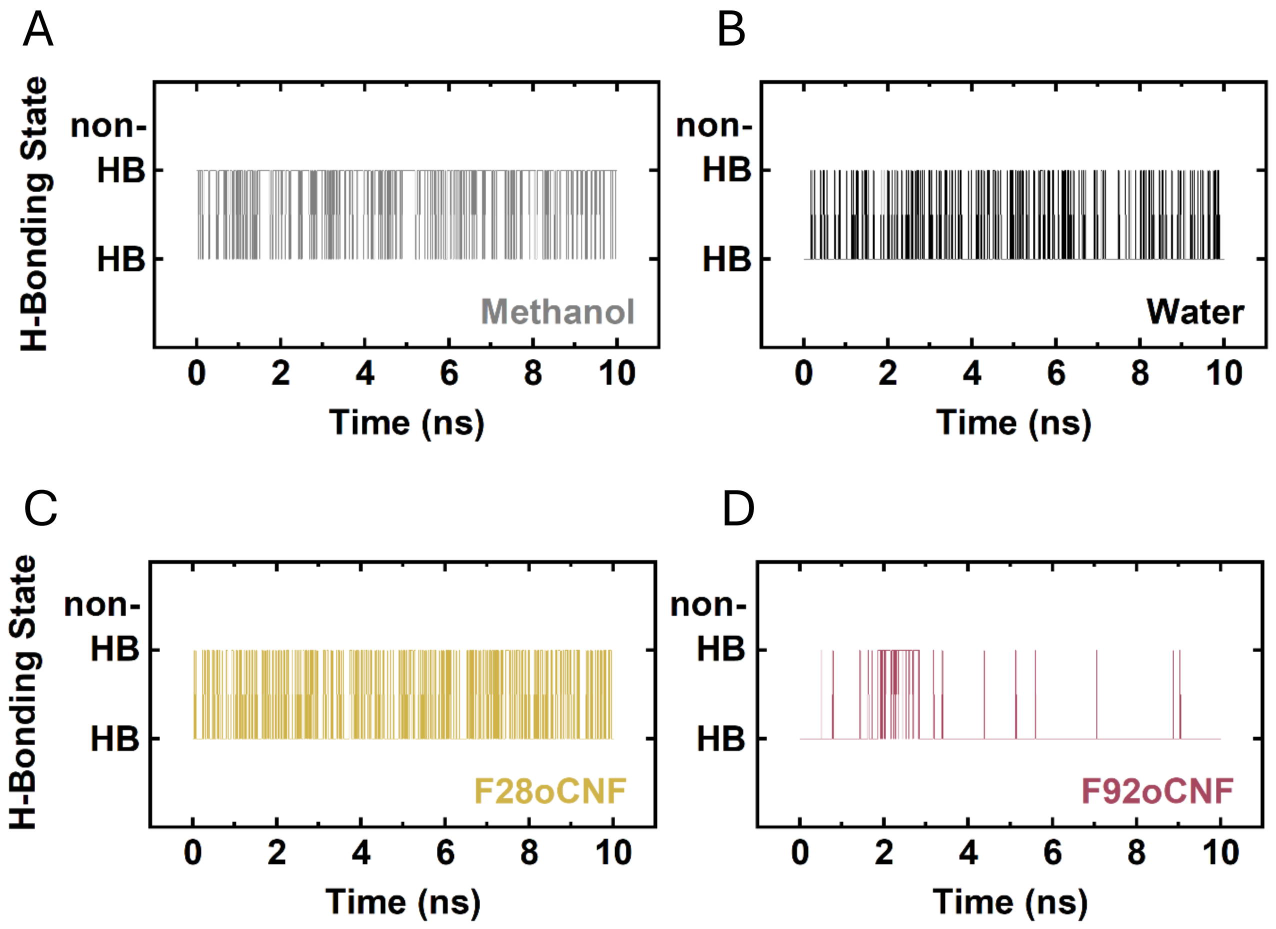

Figure S12. A, B: Analogous to Figure S11A, B but with states shown over 10 ns (rather than 1 ns); see description in Figure S11. C: In F28oCNF, a constant fluctuation between H-bonding and non-H-bonding states is observed. D: In contrast, F92oCNF's C≡N resides in a H-bonding state for extended periods (up to 1 ns), with only sporadic fluctuations into the non-H-bonding state.

11. Determination of HB blueshifts from experimental spectra

The HB blueshift $\Delta\bar{\nu}_{HB}$ (see eq. 2 in the main text) is defined as the difference between the observed vibrational wavenumber $\bar{\nu}_{obs}$ in a H-bonding environment and the pure VSE-based wavenumber $\bar{\nu}_{VSE}(\vec{F})$ due to the electric field-response of the C≡N to the electric field $\vec{F}$ of the environment (defined in *eq. S17* below):

$$\Delta\bar{\nu}_{HB} = \bar{\nu}_{obs} - \bar{\nu}_{VSE}(\vec{F}) \qquad \textit{eq. S16}$$

To determine the frequencies due to the VSE alone, the electric fields $|\vec{F}|$ exerted on the C≡N group were determined from MD simulations. In this case, it is important that MD production runs were long enough to warrant sufficient sampling of environmental dynamics and/or conformational degrees of freedom. The MD-based electric fields $|\vec{F}|$ (averaged over entire trajectories and over the relevant H-bonding fraction; see note below for additional information) are determined via induced dipoles in AMOEBA MD simulations as described in the Methods Section. Using these fields, the frequencies due to the VSE alone were determined by

$$\bar{\nu}_{VSE}(\vec{F}) = \bar{\nu}_0 - |\Delta\vec{\mu}| \cdot |\vec{F}| = 2231.2\ \text{cm}^{-1} + 0.19\ \frac{\text{cm}^{-1}}{\text{MV/cm}^{-1}} \cdot |\vec{F}| \qquad \textit{eq. S17}$$

with $\bar{\nu}_0$ = zero-field frequency and $|\Delta\vec{\mu}|$ = Stark tuning rate.

Table S10. Determination of experiment-based HB blueshifts for the C≡N group of oTN in water and methanol and of oCNF in F28oCNF and F92oCNF. Absolute AMOEBA-based electric fields ($|\vec{F}|$) were used here (see below) and determined as described in the Methods Section. $\bar{\nu}_{VSE,HB}(\vec{F})$ was calculated for the MD H-bonding fraction using eq. S17 and the parameters determined in ref. [9]. $\bar{\nu}_{obs}$ is the vibrational frequency extracted from the experimental IR spectra (see Figure 5 in the main text). In water, F28oCNF, and F92oCNF, $\bar{\nu}_{obs}$ refers to the peak maximum of the symmetric lineshape; in methanol, two bands are found, which are consistent with a H-bonding and a non-H-bonding population (see Figure S13 and Figure S14), and $\bar{\nu}_{obs}$ is provided for both. $\Delta\bar{\nu}_{HB}$ is determined according to eq. S16, using the entries for $\bar{\nu}_{VSE,HB}(\vec{F})$ and $\bar{\nu}_{obs}$.

System	$\lvert\vec{F}\rvert$ (MD, HB fraction) / MV/cm	$\bar{\nu}_{VSE,HB}(\vec{F})$ / cm^{-1}	$\bar{\nu}_{obs}$ / cm^{-1}	$\Delta\bar{\nu}_{HB}$ / cm^{-1}
MeOH (H-bonding)	-54.2	2220.9	2233.3	12.4
MeOH (non-H-bonding)	-23.5	2226.7	2227.7	(1.0)
Water	-73.0	2217.3	2231.5	14.5
F28oCNF[a]	-64.9	2219.1	2230.9	11.8
F92oCNF[a]	-78.6	2215.5	2241.3	25.8

[a] taken from ref. [14]

On the electric fields employed in this work

Note that herein, we used absolute AMOEBA-based electric fields along the C≡N. As discussed in more detail in our previous work,[14] electric fields along oTN's C≡N are easily represented as *absolute electric fields* (i.e., electric fields resulting from *all* induced dipoles in the simulation) during the MD simulations or as *environmental electric fields* due only to the solvent (i.e. intramolecular contributions due to oTN are removed by subtracting the electric field on the C≡N for oTN in vacuum). Instead, for oCNF's C≡N, only *absolute electric fields* during the MD simulations are easily determined; *environmental electric fields* due only to solvent and protein environments requires introducing 'cuts' through the oCNF sidechain (see SI Section S1 in ref. [14]). In order to avoid such complications with *environmental electric fields*, we herein stick to *absolute electric fields*. As shown in our previous work for oTN,[14] the use of absolute electric fields introduces an offset of roughly 6 MV/cm compared with environmental electric fields: this value translates to an error in the vibrational frequencies of 1.1 cm^{-1} (using eq. S17). By comparison, the HB blueshifts discussed in the main text are 10 – 25 times this value, indicating our conclusions are not affected by the electric field type.

Comment on the blueshift for oTN in MeOH

In the IR spectrum for oTN in MeOH (Figure S13), we observed an asymmetric lineshape with two distinct minima in the 2nd derivative. Fitting two Pseudo-Voigt bands to the spectrum and its 2nd derivative, we obtain two components at 2227.7 and 2233.3 cm^{-1}. Analyzing the AMOEBA MD simulations of oTN in MeOH, we also obtain two distinct distributions due to non-H-bonding and H-bonding populations (Figure S14), with average electric fields along the nitrile of -23.2 and -54.2 MV/cm, respectively. Using the VSE equation (eq. S17), these electric fields can be translated to vibrational frequencies of 2226.7 and 2220.9 cm^{-1}, respectively. As such, the 2227.7 cm^{-1} / 2226.7 cm^{-1} band can be assigned to the non-H-bonding fraction of oTN in MeOH (i.e. with a negligible HB blueshift of 1.0 cm^{-1} according to eq. S17). In that case, the 2233.3 cm^{-1} / 2220.9 cm^{-1} band can be assigned to the H-bonding fraction with a HB blueshift $\Delta\bar{\nu}_{HB}$ = 12.4 cm^{-1}. This interpretation that distinct non-H-bonding and H-bonding fractions are detected in the IR spectra is supported by the very good match between experimental and computational spectra (see Figure 6C in main text).

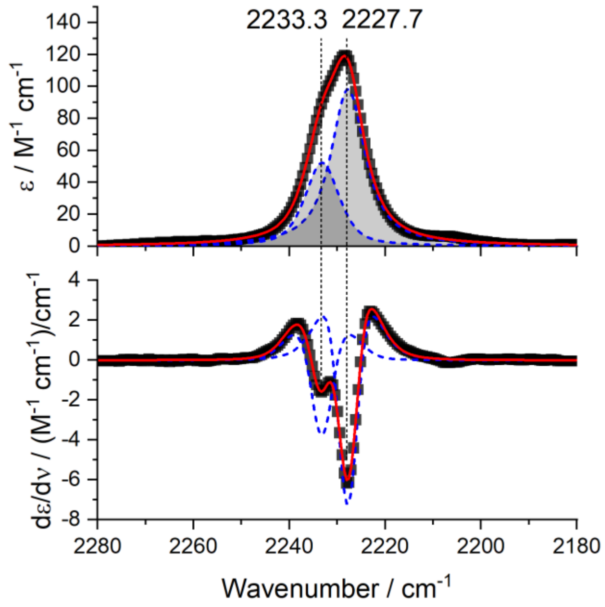

Figure S13. Simultaneous fits for the IR spectrum of oTN in methanol (top) and its 2nd derivative spectrum (bottom) to extract contributions due to H-bonding and non-H-bonding populations. Data for the experimental spectrum and its 2nd derivative are shown as squares. The absorption spectrum displays an asymmetric lineshape (top) with two minima in the 2nd derivative, which are consistent with a fit with two Pseudo-Voigt shaped bands at 2233.3 cm^{-1} (integrated area: ε = 602.8 M^{-1} cm^{-1}; full-width at half maximum: FWHM = 8.6 cm^{-1}; Gaussian fraction: n = 0.337) and 2227.7 cm^{-1} (integrated area: ε = 1280.7 M^{-1} cm^{-1}; full-width at half maximum: FWHM = 8.5 cm^{-1}; Gaussian fraction: n = 0.082). Based on comparison with Figure S14, the components at 2227.7 cm^{-1} and 2233.3 cm^{-1} are assigned to non-H-bonding and H-bonding populations, respectively.

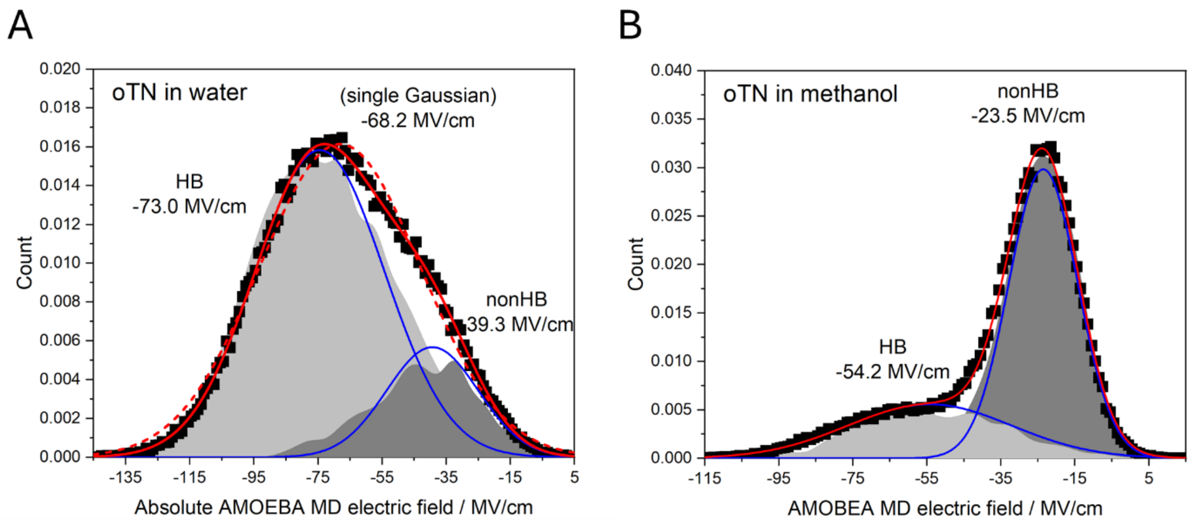

Figure S14. Distributions of absolute AMOEBA MD electric fields exerted on the C≡N for oTN in water (A) and methanol (B). Light gray areas are sub-distributions of non-H-bonding populations defined by heavy atom distances > 4.0 Å; dark gray areas are due to the H-bonding populations with heavy atom distances < 4.0 Å and N_{CN} --- D – H angles (HB cones) < 30°. Solid red lines are fits to the overall distributions using two Gaussians to extract the average electric fields for the non-H-bonding and H-bonding populations (solid blue lines). In water (B), a single Gaussian fit was also used for comparison.

12. Distributions of HB blueshifts from AMOEBA MD simulations

In Figure 5A – D in the main text, we determined average HB blueshifts $\langle \Delta\bar{\nu}_{HB}(d,\theta) \rangle$ from average HB distances and angles according to main text eq. 7. Alternatively, we also determined $\Delta\bar{\nu}_{HB}(d,\theta)$ of each MD frame, which yielded nearly normal HB blueshift distributions. Fitting Gaussians to these distributions, we determined an alternative set of average HB blueshifts (specified in Figure S15) which are highly similar to those used in the main text (within ±2 cm^{-1}).

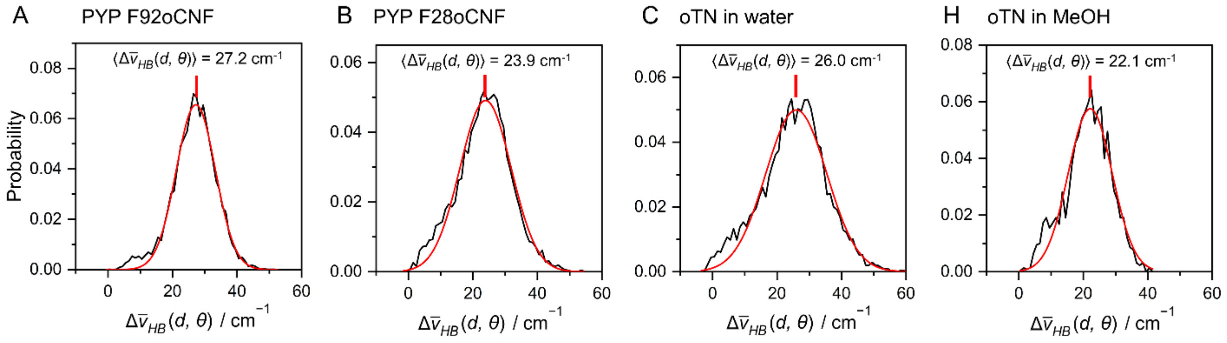

Figure S15. Predicted $\Delta\bar{\nu}_{HB}(d,\theta)$ distributions from AMOEBA MD simulations using the model and parameters in eq. 7 and Table 1 for F92oCNF (A), F28oCNF (B), oTN in water (C), and oTN in methanol (D). A HB distance threshold of 4.0 Å was applied at each MD frame to determine whether the frame was H-bonding (and its blueshift was added to the distribution) or non-H-bonding. Average values $\langle \Delta\bar{\nu}_{HB}(d,\theta) \rangle$ were obtained from fitting each distribution with a Gaussian, and R^2 values of 0.99, 0.98, 0.97, and 0.95 were obtained (A – D, respectively).

13. References

(1) Frisch, M. J.; Trucks, G. W.; Schlegel, H. B.; Scuseria, G. E.; Robb, M. A.; Cheeseman, J. R.; Scalmani, G.; Barone, V.; Mennucci, B.; Petersson, G. A.; Nakatsuji, H.; Caricato, M.; Li, X.; Hratchian, H. P.; Izmaylov, A. F.; Bloino, J.; Zheng, G.; Sonnenberg, J. L.; Hada, M.; Ehara, M.; Toyota, K.; Fukuda, R.; Hasegawa, J.; Ishida, M.; Nakajima, T.; Honda, Y.; Kitao, O.; Nakai, H.; Vreven, T.; Montgomery, J. A., Jr.; Peralta, J. E.; Ogliaro, F.; Bearpark, M.; Heyd, J. J.; Brothers, E.; Kudin, K. N.; Staroverov, V. N.; Kobayashi, R.; Normand, J.; Raghavachari, K.; Rendell, A.; Burant, J. C.; Iyengar, S. S.; Tomasi, J.; Cossi, M.; Rega, N.; Millam, J. M.; Klene, M.; Knox, J. E.; Cross, J. B.; Bakken, V.; Adamo, C.; Jaramillo, J.; Gomperts, R.; Stratmann, R. E.; Yazyev, O.; Austin, A. J.; Cammi, R.; Pomelli, C.; Ochterski, J. W.; Martin, R. L.; Morokuma, K.; Zakrzewski, V. G.; Voth, G. A.; Salvador, P.; Dannenberg, J. J.; Dapprich, S.; Daniels, A. D.; Farkas, Ö.; Foresman, J. B.; Ortiz, J. V.; Cioslowski, J.; Fox, D. J. Gaussian 16. Gaussian, Inc.,: Wallingford CT 2016.

(2) Becke, A. D. Density-functional Thermochemistry. III. The Role of Exact Exchange. *J. Chem. Phys.* **1993**, *98* (7), 5648–5652.

(3) Lee, C.; Yang, W.; Parr, R. G. Development of the Colic-Salvetti Correlation-Energy into a Functional of the Electron Density Formula. *Phys. Rev. B* **1988**, *37*, 785–789.

(4) Stephen, P. J.; Devlin, F. J.; Chabalowski, C. F.; Frisch, M. J. Ab Initio Calculation of Vibrational Absorption. *J. Phys. Chem.* **1994**, *98* (45), 11623–11627.

(5) Caldeweyher, E.; Bannwarth, C.; Grimme, S. Extension of the D3 Dispersion Coefficient Model. *J. Chem. Phys.* **2017**, *147* (3), 034112.

(6) McLean, A. D.; Chandler, G. S. Contracted Gaussian Basis Sets for Molecular Calculations. I. Second Row Atoms, Z=11-18. *J. Chem. Phys.* **1980**, *72* (10), 5639–5648.

(7) Krishnan, R.; Binkley, J. S.; Seeger, R.; Pople, J. A. Self - Consistent Molecular Orbital Methods. XX. A Basis Set for Correlated Wave Functions. *J. Chem. Phys* **1980**, *72*, 650–654.

(8) Andersson, M. P.; Uvdal, P. New Scale Factors for Harmonic Vibrational Frequencies Using the B3LYP Density Functional Method with the Triple-ζ Basis Set 6-311+G(d,p). *J. Phys. Chem. A* **2005**, *109* (12), 2937–2941.

(9) Weaver, J. B.; Kozuch, J.; Kirsh, J. M.; Boxer, S. G. Nitrile Infrared Intensities Characterize Electric Fields and Hydrogen Bonding in Protic, Aprotic, and Protein Environments. *J. Am. Chem. Soc.* **2022**, *144*, 7562–7567.

(10) Chubb, K. L.; Tennyson, J.; Yurchenko, S. N. ExoMol Molecular Line Lists – XXXVII. Spectra of Acetylene. *Mon. Not. R. Astron. Soc.* **2020**, *493* (2), 1531–1545.

(11) Schnieders, M. J.; Rackers, J. A.; Wang, Z.; Lu, C.; Laury, M. L.; Lagarde, L.; Piquemal, J.; Ren, P.; Ponder, J. W. Tinker8: Software Tools for Molecular Design. *J. Chem. Theory Comput.* **2018**, *14*, 5273–5289.

(12) Shi, Y.; Xia, Z.; Zhang, J.; Best, R.; Wu, C.; Ponder, J. W.; Ren, P. Polarizable Atomic Multipole-Based AMOEBA Force Field for Proteins. *J. Chem. Theory Comput.* **2013**, *9* (9), 4046–4063.

(13) Ren, P.; Wu, C.; Ponder, J. W. Polarizable Atomic Multipole-Based Molecular Mechanics for Organic Molecules. *J. Chem. Theory Comput.* **2011**, *7* (10), 3143–3161.

(14) Kirsh, J. M.; Weaver, J. B.; Boxer, S. G.; Kozuch, J. Critical Evaluation of Polarizable and Nonpolarizable Force Fields for Proteins Using Experimentally Derived Nitrile Electric Fields. *J. Am. Chem. Soc.* **2024**, *146*, 6983–6991.

(15) Zheng, C.; Mao, Y.; Kozuch, J.; Atsango, A. O.; Ji, Z.; Markland, T. E.; Boxer, S. G. A Two-Directional Vibrational Probe Reveals Different Electric Field Orientations in Solution and an Enzyme Active Site. *Nature Chem.* **2022**, *14*, 891–897.

(16) Ji, Z.; Kozuch, J.; Mathews, I. I.; Diercks, C. S.; Shamsudin, Y.; Schulz, M. A.; Boxer, S. G. Protein Electric Fields Enable Faster and Longer-Lasting Covalent Inhibition of β-Lactamases. *J. Am. Chem. Soc.* **2022**, *144* (45), 20947–20954.

(17) Kozuch, J.; Schneider, S.; Zheng, C.; Ji, Z.; Bradshaw, R. T.; Boxer, S. Testing the Limitations of MD-Based Local Electric Fields Using the Vibrational Stark Effect in Solution: Penicillin G as a Test Case. *J. Phys. Chem. B* **2021**, *125*, 4415–4427.

(18) Wang, Z.; Ponder, J. W. *Tinker9: Next Generation of Tinker with GPU Support*. https://github.com/TinkerTools/tinker9.

(19) Walker, B.; Liu, C.; Wait, E.; Ren, P. Automation of AMOEBA Polarizable Force Field for Small Molecules: Poltype 2. *J. Comput. Chem.* **2022**, *43* (23), 1530–1542.

(20) Auer, B. M.; Skinner, J. L. Dynamical Effects in Line Shapes for Coupled Chromophores: Time-Averaging Approximation. *J. Chem. Phys.* **2007**, *127*, 104105.

(21) Schmidt, J. R.; Corcelli, S. A. Infrared Absorption Line Shapes in the Classical Limit: A Comparison of the Classical Dipole and Fluctuating Frequency Approximations. *J. Chem. Phys.* **2008**, *128* (18).

(22) Fried, S. D.; Bagchi, S.; Boxer, S. G. Measuring Electrostatic Fields in Both Hydrogen Bonding and Non-Hydrogen Bonding Environments Using Carbonyl Vibrational Probes. *J. Am. Chem. Soc.* **2013**, *135* (30), 11181–11192.

(23) Kirsh, J. M.; Weaver, J. B.; Boxer, S. G.; Kozuch, J. Critical Evaluation of Polarizable and Nonpolarizable Force Fields for Proteins Using Experimentally Derived Nitrile Electric Fields. *J. Am. Chem. Soc.* **2024**, *146*, 6983–6991.

(24) Weaver, J. B.; Kozuch, J.; Kirsh, J. M.; Boxer, S. G. Nitrile Infrared Intensities Characterize Electric Fields and Hydrogen Bonding in Protic, Aprotic, and Protein Environments. *J. Am. Chem. Soc.* **2022**, *144*, 7562–7567.

(25) Ji, Z.; Mukherjee, S.; Andreo, J.; Sinelschikova, A.; Peccati, F.; Wuttke, S.; Boxer, S. G. Electrostatic Atlas of Noncovalent Interactions Built in Metal-Organic Frameworks. *ChemRxiv* **2024**, DOI: 10.26434/chemrxiv-2024-xfrjs.

(26) Fried, S. D.; Boxer, S. G. Measuring Electric Fields and Noncovalent Interactions Using the Vibrational Stark Effect. *Acc. Chem. Res.* **2015**, *48*, 998–1006.

(27) Cruz, R.; Ataka, K.; Heberle, J.; Kozuch, J. Evaluating Aliphatic CF, CF2 and CF3 Groups as Vibrational Stark Effect Reporters. *J. Chem. Phys.* **2024**, *160*, 204308.

(28) Dalosto, S. D.; Vanderkooi, J. M.; Sharp, K. A. Vibrational Stark Effects on Carbonyl, Nitrile, and Nitrosyl Compounds Including Heme Ligands, CO, CN, and NO, Studied with Density Functional Theory. *J. Phys. Chem. B* **2004**, *108* (20), 6450–6457.

(29) Cruz, R.; Ataka, K.; Heberle, J.; Kozuch, J. Evaluating Aliphatic CF, CF2 and CF3 Groups as Vibrational Stark Effect Reporters. *J. Chem. Phys.* **2024**, *160*, 204308.

(30) Karssemeijer, L. J.; Pedersen, A.; Jónsson, H.; Cuppen, H. M. Long-Timescale Simulations of Diffusion in Molecular Solids. *Phys. Chem. Chem. Phys.* **2012**, *14* (31), 10844–10852.
